# Integrative Network Analysis of Single-Cell RNA Findings and a Priori Knowledge Highlights Gene Regulators in Multiple Myeloma Progression

**DOI:** 10.3390/ijms27020793

**Published:** 2026-01-13

**Authors:** Grigoris Georgiou, Margarita Zachariou, George M. Spyrou

**Affiliations:** Bioinformatics Department, The Cyprus Institute of Neurology & Genetics, 6 International Airport Avenue, 2370 Nicosia, Cyprus; grigorisg@cing.ac.cy (G.G.); margaritaz@cing.ac.cy (M.Z.)

**Keywords:** Multiple Myeloma, plasma cells, malignancy, cancer, scRNA, network analysis, regulation, network integration

## Abstract

Multiple Myeloma (MM) is an incurable malignancy that progresses from asymptomatic precursor stages—Monoclonal Gammopathy of Undetermined Significance (MGUS) and Smouldering Multiple Myeloma (SMM)—to active disease. Despite ongoing research, the molecular mechanisms driving this progression remain poorly understood. In this study, we aimed to uncover key regulatory factors involved in MM progression by integrating single-cell RNA sequencing (scRNA-seq) data with curated a priori biological knowledge of MM. To this end, we first integrated a priori knowledge from databases in a synthetic gene network map to play the role of an MM-related backbone to project findings from scRNA analysis on CD138^+^ Plasma Cells. This was followed by stage-specific regulatory network construction and analysis using Integrated Value of Influence (IVI) metrics to identify the most influential genes across disease stages. Our findings revealed *GSK3B*, *RELA*, *CDKN1A*, and *PCK2* as central regulators shared across multiple stages of the disease. Notably, several of these genes had not previously been included in established MM gene sets, highlighting them as prime candidates for biomarkers and drug targets.

## 1. Introduction

Multiple Myeloma (MM) is the second most prevalent haematological malignancy, with significant biological and genetic heterogeneity [1]. It predominantly affects older adults, where the mean and median ages at diagnosis range from 70 to 73 years, with 63% of patients being over the age of 65, and accounts for more than 10% of hematologic cancers and approximately 1% of all cancers [2,3]. The disease is marked by the proliferation of abnormal plasma cells (PCs) in the bone marrow (BM) and elevated levels of monoclonal protein in the blood and urine [4], which are key features used to classify patients across the premalignant and asymptomatic stages, namely Monoclonal Gammopathy of Undetermined Significance (MGUS) and Smouldering Multiple Myeloma (SMM), to active MM.

MGUS is typically detected incidentally during routine laboratory investigations, affecting approximately 2.4% of the general population. A subset of MGUS patients progresses to MM or related disorders at an estimated rate of 1% per year [5]. SMM represents a heterogeneous patient group with biological characteristics resembling MGUS but carries a higher risk of progression to active MM. It is estimated that 51% of SMM patients will progress to active MM within 5 years of diagnosis, increasing to 66% within 10 years. Given the variability in progression risk among SMM patients, those classified as high-risk allow for tailored disease management strategies [6].

Despite significant improvements in patient prognosis over recent decades, driven by therapeutic advances such as proteasome inhibitors (PIs), including immunomodulatory agents (IMID), monoclonal antibodies, bispecific antibodies, and chimeric antigen receptor T-cell (CAR-T) immunotherapy, MM remains incurable, primarily due to inevitable relapse and the development of drug resistance during maintenance therapy [7]. Ho et al. (2020) noted that there is no recommended treatment for MGUS or SMM; instead, a “watch-and-wait” strategy is generally employed, with bisphosphonates optionally considered for SMM patients [8]. According to Savva et al. (2025), current management of the two premalignant stages includes regular monitoring for MGUS and low-risk SMM, which avoids drug toxicity but carries a small ongoing risk of progression, while in high-risk SMM, early treatment can delay progression and improve outcomes [9]. This challenge is further compounded by the limited information available in publicly accessible databases for these stages. Therefore, understanding the regulatory mechanisms underlying disease progression is essential for identifying potential interventions capable of reversing or halting this process.

In this regard, several computational and omics-based studies have sought to unravel the molecular mechanisms underlying MM progression. For instance, Saadoune et al. (2022) [10] compiled a list of 114 differentially expressed genes (DEGs) associated with MM prognosis from various scientific publications and databases. By employing a network-based approach—including protein–protein interaction (PPI) networks, co-expression analysis, and network analytics—they identified seven core genes with significant roles in MM, namely *TP53*, *MYC*, *CCND1*, *IL6*, *UBA52*, *EZH2*, and *MDM2* [10].

Building on the concept of an integrative multisource network-driven approach, we collected and curated comprehensive knowledge on MM from publicly available databases, forming a robust gene backbone representing MM current a priori knowledge. To further enhance this dataset, we integrated it with two single-cell RNA sequencing (scRNA-seq) datasets containing CD138^+^ PCs [11]. Single-cell analytics offer high-resolution insights not only at the cell type level but also across disease stages, enabling the mapping of the cellular and molecular landscape of active MM as well as its precursor stages, MGUS and SMM [12]. When combined with structured a priori knowledge, they provide a powerful framework for dissecting the mechanisms that drive disease progression.

**Figure 1:**
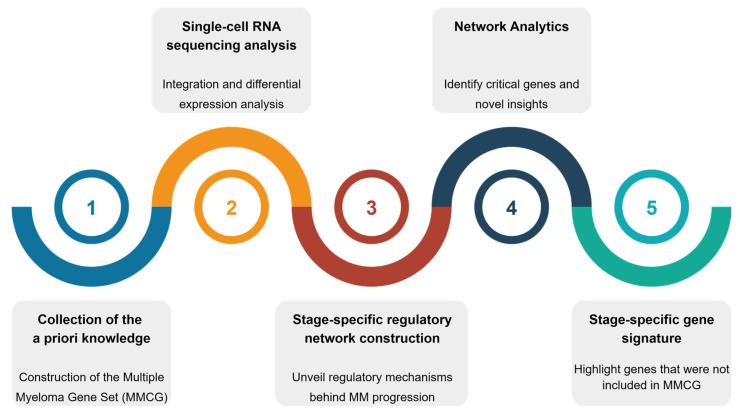
Overview of the study. The pipeline starts with the collection of Multiple Myeloma (MM)-related information from multiple sources, followed by integration and differential expression analysis of CD138^+^ single-cell RNA sequencing (scRNA-seq)data. Stage-specific regulatory networks are then constructed, and network analytics are applied to rank key genes. The workflow concludes with the identification of common and stage-specific gene signatures.

By combining these approaches, we constructed stage-specific regulatory networks to capture key molecular interactions across MGUS, SMM, and MM, identifying critical genes and novel insights into MM. This integrative framework, shown in Figure 1, provides a deeper understanding of the regulatory landscape of MM and uncovers genes that may play critical roles in its development.

## 2. Results

### 2.1. Collection of the a Priori Knowledge Highlights Key Disease-Related Genes

We recall that this study aimed to uncover the regulatory mechanisms underlying active MM and its precursor stages (MGUS and SMM) by integrating single-cell analytics with a priori biological knowledge. The rationale behind this approach was the assumption that, by systematically collecting and organising existing knowledge, we could better identify what remains unknown or has been overlooked in recent experimental studies.

To this end, we began by searching for publicly available information specific to each disease stage. However, our investigation revealed that curated data for the precursor stages MGUS and SMM were extremely limited. Therefore, we focused on compiling comprehensive information related to active MM, collecting data across five knowledge categories: Drugs, miRNAs, Variants, Genes, and Proteins.

**Figure 2:**
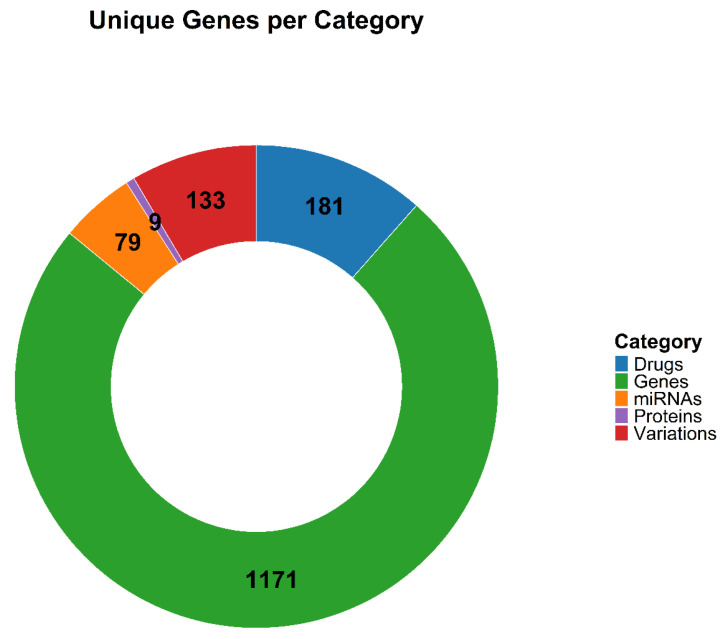
Number of Associated Genes in each source category. This doughnut chart shows the number of associated genes in each of the five categories.

**Figure 3:**
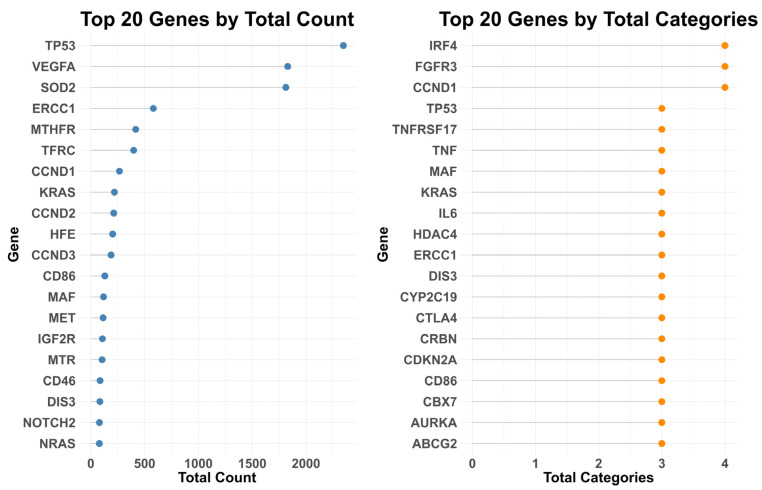
Top 20 Genes Based on Total Counts and Category Coverage. This lollipop plot displays the top 20 genes with the strongest representation in the MM Core Gene set (MMCG set), ranked by total number of occurrences (Total Counts) and the number of categories in which they appear (Total Categories).

This process resulted in a curated set of 1412 genes, with the majority originating from the “Genes” category (Figure 2 and Supplementary File S2). In contrast, the “Proteins” category contained the least information, comprising only nine genes. To quantify the relevance and breadth of representation for each gene, we calculated two metrics: (1) the total number of times each gene appeared across the five categories, and (2) the total number of categories in which each gene was represented (Supplementary File S2). These metrics helped highlight the most prominent and well-defined genes in MM—those that were both frequently mentioned and broadly distributed across knowledge types (Figure 3).

According to these calculations, *TP53* [13,14], *VEGFA* and *SOD2* ranked as the top three genes based on their total number of occurrences across the five categories, followed by *CCND1* [13,15,16,17], *KRAS* [18,19,20] and *CCND2* [16], both of which are among the most extensively studied genes in MM. *CCND1* was also found in four out of five categories, along with *IRF4* [15,16,18,20] and *FGFR3* [21,22], which are likewise well known for their roles in MM biology.

### 2.2. Single-Cell RNA Analysis Highlights Key Stage-Specific Disease-Related and Immunoglobulin Structure-Related Genes

**Figure 4:**
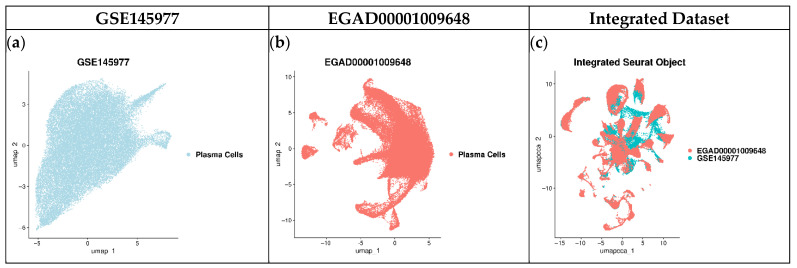
scRNA-seq datasets UMAP representation. UMAP from classical scRNA analysis represents each dataset individually and the integrated dataset. Colours indicate clusters of plasma cells. The integrated UMAP demonstrates that batch correction was successful.

While previous studies provided valuable insights at the bulk level, single-cell approaches enable the resolution of cellular heterogeneity and transitional states that are critical in disease evolution. Based on this, two scRNA-seq datasets, each profiling CD138^+^ PCs, a key marker of MM progression, were integrated to increase statistical power and sample diversity, enabling a more comprehensive characterisation of transcriptional changes across MM stages (Figure 4).

**Figure 5:**
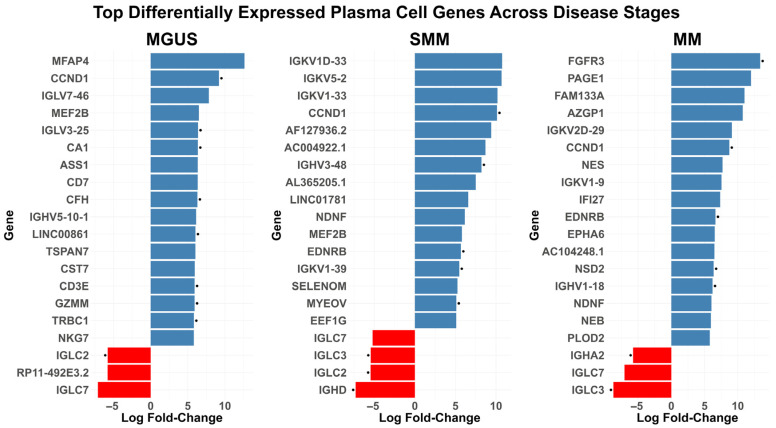
Top 20 Differentially Expressed Genes (DEGs) based on absolute log fold change (LogFC). Bar plots illustrating the top 20 DEGs ranked by the mean of absolute LogFc for each disease stage. Genes are sorted by absolute LogFc, with bars colour-coded to represent the direction of regulation: upregulated genes are depicted in blue, while downregulated genes are shown in red. Dot presence indicates the availability of the represented gene in the MMGC.

The Differential Expression Analysis (DEA) of the Integrated Dataset, presented in Figure 5, highlighted a variety of genes from the MMCG. Notably, *CCND1* was overexpressed across all three stages, while FGFR3 was overexpressed only in the MM stage. Beyond these two genes, the top 20 DEGs based on absolute LogFC included genes related to immunoglobulin structure like *IGLC2*, *IGLC3*, *IGLV3-25*, *IGHD* and *IGHA2*, also present in the MMCG. The identification of these well-established MM genes following the DEA provided a validation of the effectiveness of our single-cell integration strategy in capturing key regulators of MM biology.

### 2.3. Regulatory Network Construction and Analysis Highlights Stage-Specific Influential Genes

Building on the findings from scRNA analysis, we proceeded with the construction of regulatory networks to further investigate the molecular mechanisms behind these stage-specific CD138^+^ PCs. After filtering the final DEA lists based on p_adj_ and logFC, and incorporating genes from the MMGC that passed the initial p_adj_ filtering, we performed stage-specific queries using the downloaded Signor database. During this process, some genes were excluded either because they were not yet included in Signor or lacked any relationships with other genes. Additionally, since the Signor database contains information from various sources and organisms, the results were further filtered to retain only entries where UniProt was the source and Homo sapiens was the organism. The stage-specific regulatory networks (Figures S1–S3), served as the foundation for subsequent network comparisons and analyses. Network analysis was performed on each regulatory network by calculating both the in-, out- and all-IVI scores of each node and its in-, out- and all-degree (Figures S4–S12). The top-ranked genes for each stage based on their all-IVI scores are shown in Table 1.

**Figure 6:**
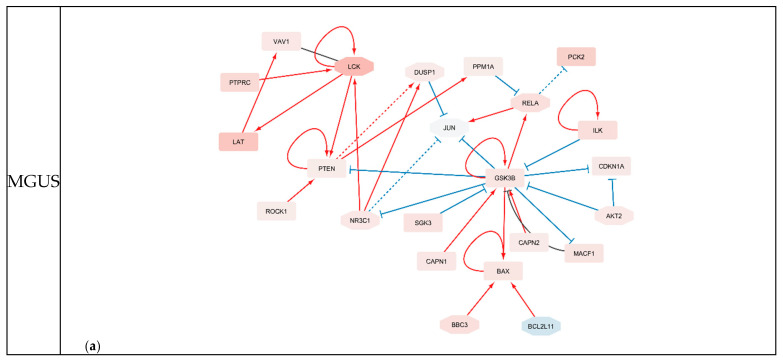

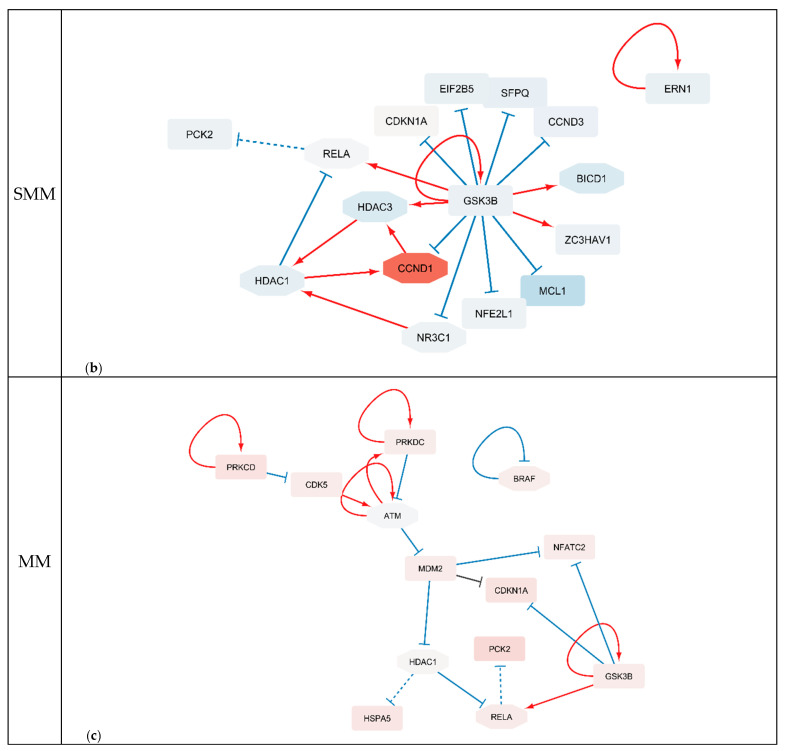
Stage-specific gene regulatory relationships. This figure illustrates the regulatory interactions between genes identified for each disease stage. Node colour represents the stage-specific LogFCscaled from blue (downregulated) to red (upregulated). Nodes with an octagonal shape indicate genes derived from the MMCG dataset. Edges are coloured according to regulatory effect: red for activation, blue for inhibition, and black for undefined effects. Edge direction and style further reflect regulatory type—standard arrows denote activation, T-shaped arrows indicate inhibition, dotted edges signify that the interaction leads to transcriptional activation or repression of the target gene, and vertical slash edges signify that the interaction occurs due to binding of their proteins that lead to either activation or inactivation.

Table 2 presents the full lists of highlighted genes for each MM stage, using a threshold of IVI ≥ 25 for IVI_in, IVI_out, and IVI_all. According to these results, *NR3C1* was underscored in both MGUS and SMM; *HDAC1* in SMM and MM; and *RELA*, *GSK3B*, *CDKN1A* and *PCK2* were consistently identified across all three stages. From those genes stated above, only *GSK3B*, SGK3, CDKN1A and PCK2 were not included in the MMCG. In addition, Table 3 summarises the genes identified by our analysis that were not part of the MMCG, together with their corresponding IVI scores, including stage-specific and shared candidates. Finally, based on the results shown in Table 2, new regulatory networks were constructed to illustrate the regulatory relationships among the highlighted genes (Figure 6).

## 3. Discussion

Multiple Myeloma is an incurable blood malignancy. The progression from MGUS and SMM to active MM is not well understood. This knowledge gap is particularly concerning given the lack of effective therapeutic interventions for MGUS and SMM, and the eventual development of treatment resistance in most MM patients. Understanding the mechanisms that drive this progression is therefore crucial for identifying early intervention targets that could slow, halt, or even reverse disease development.

To achieve this, we first collected and aggregated curated information from multiple publicly available databases, focusing on five categories: Genes, miRNAs, Proteins, Variants, and Drugs. Due to limited curated data for MGUS and SMM, we focused on MM-specific resources, generating the MMCG set as a reference to highlight genes and mechanisms that may have been previously overlooked. As expected, established MM-associated genes, such as *CCND1*, *CCND2*, *TP53*, *KRAS*, *IRF4*, and *FGFR3*, were highlighted in the initial MMCG analysis. These genes have previously been validated for their involvement in MM through metaphase cytogenetics, fluorescence in situ hybridisation (FISH), and high-throughput sequencing approaches, including Whole-Exome Sequencing and Whole-Genome Sequencing [14].

To further refine the analysis and increase resolution, we integrated data from two scRNA-seq datasets of CD138^+^ PCs, as CD138 serves as a key molecular marker of PCs, with its expression restricted to terminally differentiated B cells [23]. The selection of this antigen was based on its critical role in MM, as the loss of its function has been shown to impair MM cell growth in vitro and trigger apoptosis in vivo, highlighting its importance in maintaining cell survival and disease progression [11].

The integration of data from the MMGC and scRNA-seq analyses enabled the construction of stage-specific regulatory networks. These networks were evaluated using various network metrics, including in-degree, out-degree, total degree, as well as in-, out-, and total IVI scores to identify key regulators and targets. This was followed by the identification of the most influential nodes across disease stages. After applying filtering criteria, we obtained a short list of stage-specific candidate genes (Table 2) and focused our discussion on those not included in the original MMGC resource (Table 3), beginning with the genes common to all three conditions (*GSK3B*, *CDKN1A*, and *PCK2*).

GSK3B is a negative regulator of glucose homeostasis and plays critical roles in energy metabolism, inflammation, ER stress, mitochondrial dysfunction, and apoptotic pathways [24]. Our analysis identified *GSK3B* as a top regulatory and influential gene across all stages. A 2020 study aimed to identify genes overexpressed in myeloma stem cells that could serve as predictors for risk stratification in newly diagnosed MM patients using Ingenuity Pathway Analysis (IPA), highlighted *GSK3B*, along with *ROCK1*, also identified in our analysis, as key components of the MMSP5 gene model, both demonstrating stronger prognostic power than standard clinical parameters [25]. More recently, Zeng et al. (2023) highlighted *GSK3B* as a potential target of daucosterol in the treatment of MM [26].

*CDKN1A*, is a negative regulator of the cell cycle and, when overexpressed, leads to cell cycle arrest [27]. A 2021 study investigating DEGs and differentially expressed miRNAs in MM identified *CDKN1A* as a potential seed gene for prognosis, warranting further investigation when compared to normal PCs [28]. Another study highlighted *CDKN1A* as an upregulated hub gene in MM, based on protein–protein interaction networks constructed using 208 upregulated and 550 downregulated genes [29].

*PCK2*, a mitochondrial phosphoenolpyruvate carboxykinase involved in the first rate-limiting step of gluconeogenesis, has no established link to MM biology. However, Papanagnou et al. (2018) reported its upregulation in peripheral blood mononuclear cells from MM patients treated with proteasome inhibitors, as part of a broader stress-response programme [30]. More recently, Wang et al. (2025) [31] reported elevated *PCK2* expression across several cancer types. In diffuse large B-cell lymphoma, their functional analysis revealed that silencing PCK2 inhibited cell proliferation and induced apoptosis under low-glucose conditions [31]. Human Protein Atlas (HPA) (https://www.proteinatlas.org/) also confirms *PCK2* expression in myeloma cell lines, supporting its potential relevance in MM [32].

With respect to the stage-specific highlighted genes, starting from MGUS, *SGK3* is a member of the AGC family of serine/threonine kinases. A study by Hausmann et al. (2015) [33] investigated the potential of *SGK3* as a therapeutic target in MM by examining its role in oncogenic signalling, either independently or in cooperation with Akt, using three MM cell lines. Their findings showed that *SGK3* depletion, alone or in combination with Akt inhibition, did not significantly affect the survival or viability of MM cells, suggesting a limited *SGK3*-dependent contribution in these models. However, the study lacked functional analyses using primary MM cells. The authors noted that *SGK3* inhibition could still hold therapeutic value in clinical settings or specific MM subgroups [33].

Furthermore, the role of *MACF1* is in MM biology has not yet been explored. *MACF1* has been previously implicated in the Wnt/β-catenin signalling pathway and is closely associated with the Axin complex, which includes Axin, β-catenin, *GSK3B*, and *APC*. In cancer, *MACF1* has been shown to promote the expression of Wnt-responsive genes, contributing to cell proliferation, migration, and signal transduction [34]. According to the HPA, *MACF1* was expressed across 34 myeloma cell lines, with protein expression confirmed in 12 of them [32].

Moreover, Calpain-1 (*CAPN1*) and Calpain-2 (*CAPN2*) are heterodimeric proteases composed of a common small regulatory subunit (CAPNS1) and distinct catalytic subunits. Both have been proposed as potential therapeutic targets in cancer due to their roles in cell-signalling pathways that affect drug sensitivity and promote metastasis [35]. In the context of MM, the only known association was reported by Zhan et al. (2003), who identified *CAPN2* expression in CD138-enriched BMPCs; however, this finding was not further explored in their study [36].

As for *ILK*, early studies in 2012 explored its potential role in MM by inhibiting and knocking down its expression in both primary MM cells and cell lines. Results showed that *ILK* could not be considered as a viable therapeutic target in MM. However, they noted that the MM microenvironment might depend on *ILK* differently than established MM cell lines [37]. More recently, Zhao et al. (2018) [38] investigated the role of *ILK* in mesenchymal stem cells (MSCs) derived from MM patients and suggested that *ILK* may contribute to MSC differentiation into smooth muscle cells through increased *α-SMA* expression. However, the underlying molecular mechanism remains undetermined [38].

Alimohammadi et al. (2024) [39] described *PTEN* as a tumour suppressor gene that inhibits the PI3K/Akt pathway, and its mutation, inactivation, or oncogene amplification contributes to MM cell formation and growth. Early evidence indicates that *PTEN* loss occurs in 5.6% of MM patients and has only been observed in those with severe illness, suggesting a late-onset event [39]. A study by Papadimitriou et al. (2023) also mentioned that miR-25-targeting of *PTEN* has been demonstrated in vitro to activate PI3K/AKT pathway, resulting in MM proliferation and apoptosis attenuation [40].

A study by Kamal et al. (2025) [41] examined *PTPRC*, also known as *CD45*, and reported that the progressive loss of CD45 pos/CD138 low and CD45 low/CD138 low PC clusters, which were prevalent in control and precursor samples, but significantly diminished with MM advancement. Based on these findings, they mentioned that early PC subtypes may either evolve into unique phenotypes or be selectively eliminated as the disease progresses [41].

Although studies on *VAV1* in PCs and MM are limited, HPA data show *VAV1* RNA expression in 34 MM cell lines, with the highest levels in NCI-H929, and proteomic analyses confirm substantial VAV1 protein expression in MM cell lines [32].

Moving to the SMM stage-specific genes, starting with *EIF2B5*, no established association with MM based on the currently available literature was found. However, data from the HPA confirmed its expression through RNA-seq and proteomic analyses in MM cell lines [32]. The potential importance of the *EIF2* signalling pathway in MM was highlighted by López-Corral et al. (2014), who identified it as one of the most significantly deregulated canonical pathways in their analysis [42].

Moreover, the *ERN1* and *HSPA5* (found in MM analysis) expressions were found to be regulated by the unfolded protein response (UPR) pathway. Those genes, together with *PRDM1* and *XBP1*, are essential for the terminal differentiation of B cells into PCs. The activation of UPR by ER stress has been linked to the transition of mature surface Ig-dependent B cells to Ig-secreting PCs that no longer express Ig on the surface. *HSPA5* seems to play an important role in this maturation, while ERN1 has been previously found highly expressed in PCs-derived MM and shown to be required during V(D)J recombination at the transition from pro- to pre-B cells [43].

*NFE2L1*, also known as *NRF1*, is a gene known for its association with MM. Previously, Jindrich Sedlacek (2025) investigated the impact of proteostasis in MM, highlighting the role of *NFE2L1* in the bounce-back response, a recovery pathway driven by *NFE2L1*, which contributes to maintaining proteasome activity via proteasome subunit resynthesis upon impairment of its function [44].

*PSIP1* encodes several splice variants of LEDGF/p75, a protein whose interactions through its PWWP domain are linked to DNA damage repair, mRNA splicing, chromatin remodelling, and transcription. Additionally, interactions through its IBD domain with oncogenic transcription factors have been associated with the upregulation of gene pathways involved in cancer progression and aggressiveness. Targeting LEDGF/p75 with single-agent inhibitors or in combination with other therapeutic compounds represents a potential avenue for treating MM, according to Ortiz-Hernandez et al. [45].

*SFPQ* was also highlighted in our analysis. According to SIGNOR, *SFPQ* is inactivated by *GSK3B* while forming a complex with NONO (Non-POU Domain Containing Octamer Binding). This complex was the focus of a study by Laurenzi et al. (2022), which demonstrated that RNA-mimetics capable of selectively binding to the RNA recognition sites of NONO and/or *SFPQ* could disrupt paraspeckle formation, thus representing a first step towards the discovery of drugs for MM treatment [46].

ZC3HAV1, a PARP family enzyme, has been shown in pancreatic cancer to regulate cell cycle progression by modulating cyclin D1 and CDK2, and to bind KRAS, enhancing its expression and activating ERK signalling to promote proliferation and metastasis [47]. Although these findings derive from another cancer type, both cell cycle control and KRAS signalling are dysregulated in MM, making *ZC3HAV1* a promising gene for further investigation in this context.

Lastly, six MM stage-specific genes were identified. For *CDK5*, Tang et al. (2020) [48] reported elevated expression in primary MM cells, cell lines, and BM biopsies, with siRNA-mediated knockdown significantly reducing MM cell viability. Pharmacological *CDK5* inhibition caused G2/M arrest and reduced *CDC25C*, *CDC2*, and cyclin B1 levels, while cyclin D1 and cyclin E1 (G1/S regulators) were unaffected [48].

*PRKCD* has been linked to several cancers, but its role in MM is unclear. Liu et al. (2020) showed that circITCH, downregulated in MM, restores bortezomib (BTZ) sensitivity via the miR-615-3p/PRKCD axis, with circITCH overexpression enhancing BTZ response [49].

*PRKDC*, primarily involved in cell cycle regulation and DNA repair, also regulates spliceosome and RNA-transport pathways, consistent with MDMS8 biology in newly diagnosed MM patients [50].

MDM2 is well-established in MM; Jovanović et al. (2018) showed that it suppresses p53 by promoting its degradation, forming a negative feedback loop, and that *MDM2* inhibition may reactivate p53 and provide therapeutic benefit [51]. Finally, the role of *NFATC2* in MM remains unknown, but findings from other cell types by Yang et al. (2020) and Schütt et al. (2021) underscore its relevance for further study [52,53].

Our study has both strengths and limitations. A key limitation was the small availability of curated data for the precursor stages, which restricted our a priori knowledge base mainly to active MM and may have reduced stage-specific resolution. However, many MMCG genes are known to participate in early genetic events preceding MM onset, supporting the biological relevance of our findings. This is a computational study, and we recognise the absence of direct experimental validation regarding our findings. However, the strength of our approach lies in the fact that many of the genes included in the MMCG have previously been experimentally validated, and several of our identified candidates have already been tested—such as in MM cell lines—providing additional confidence and biological relevance to our results.

## 4. Materials and Methods

**Figure 7:**
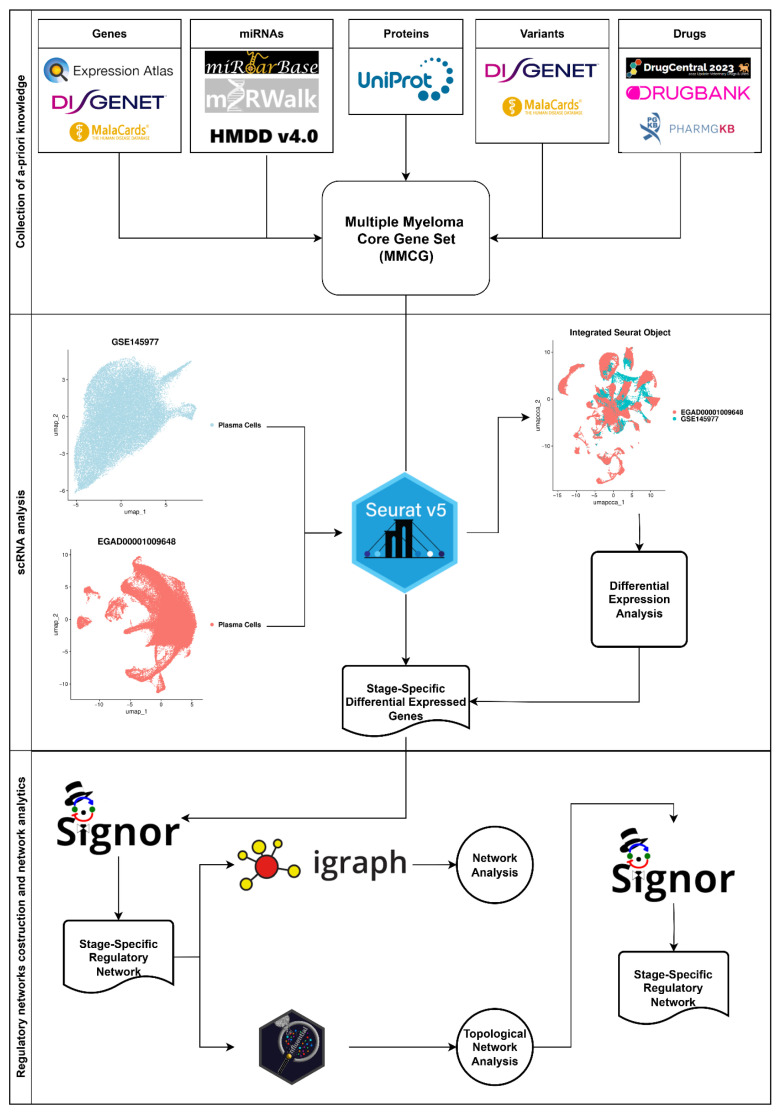
Workflow overview. The analytical workflow is divided into three main stages: (1) compilation of a priori knowledge related to MM, (2) scRNA-seq analysis involving the integration of two CD138^+^ datasets and subsequent characterisation of the integrated data, and (3) construction of stage-specific regulatory networks informed by the previous stages. Network analysis was performed by computing in-degree, out-degree and all-degree using the *igraph (version 2.2.0) R* package, as well as IVI scores (IVI_in, IVI_out, and IVI_all) using the *influential (version 2.2.9) R* package. As the degree is incorporated into IVI calculation, final gene filtering was based solely on IVI metrics. Refined stage-specific regulatory networks were then reconstructed to highlight interactions among the most influential genes.

The workflow presented in Figure 7 summarises the steps performed in this research project. The process begins with collecting a priori knowledge on MM using five key categories: genes, miRNAs, proteins, variants, and drugs. Information for each pillar was obtained from various biological databases, and all associated gene data were consolidated into an MM-specific a priori knowledge gene pool, referred to as MM Core Gene set (MMCG set). Next, scRNA analysis was conducted to investigate these genes using an integrated scRNA dataset derived from two experiments: EGAD0001009648 and GSE145977. DEA was then performed, and genes were filtered based on the adjusted *p*-value (p_adj_). Stage-specific regulatory networks were subsequently constructed using Signor 3.0 [54]. These networks were analysed using the Influential (version 2.2.9) [55] and igraph (version 2.2.0) [56] packages in R.

### 4.1. Data Sources

#### 4.1.1. Collection of the a Priori Knowledge

To construct the MMCG set, we integrated publicly available data from online databases using five categories: genes, miRNAs, proteins, variants, and drugs. For genes, data were collected from three databases: DISGENET (https://disgenet.com/, accessed on 23 May 2025) [57], Expression Atlas (https://www.ebi.ac.uk/gxa/home, accessed on 23 May 2025) [58], and Malacards (https://www.malacards.org/, accessed: 23 May 2025) [59]. For miRNAs, associated genes were collected from miRTarBase (https://awi.cuhk.edu.cn/~miRTarBase/miRTarBase_2025/php/index.php, accessed on 23 May 2025) [60], miRWalk (http://mirwalk.umm.uni-heidelberg.de/, accessed: 23 May 2025) [61], and HMDD (http://www.cuilab.cn/hmdd, accessed on 23 May 2025) [62]. Proteins were retrieved from UniProt (https://www.uniprot.org/, accessed on 23 May 2025) [62], and for variants, data were sourced from DISGENET (https://disgenet.com/, accessed on 23 May 2025) and Malacards (https://www.malacards.org/, accessed on 23 May 2025). Finally, approved drugs for MM were collected, and their targets were identified using DrugCentral (https://drugcentral.org/, accessed on 23 May 2025) [63], DrugBank (https://go.drugbank.com/, accessed on 23 May 2025) [64], and PharmGKB (https://www.pharmgkb.org/, accessed on 23 May 2025) [65]. All collected data are provided in the Supplementary Files S3–S7, while filtering criteria applied to each database are presented in Table S1.

#### 4.1.2. Single-Cell RNA Datasets

The data under study have been collected from the Gene Expression Omnibus (GEO) database (https://www.ncbi.nlm.nih.gov/geo/, accessed on 8 January 2024) (GSE145977) [66] and the European Genome-Phenotype Archive (EGA) (https://ega-archive.org/, accessed on 8 January 2024) (EGAD0001009648) [67]. Both datasets included samples from all the generic (MGUS, SMM, and MM) stages of MM, as shown in Table 4. To include only cells derived from PCs, EGAD00001009648 was split based on the presence of the CD138 antigen, and samples containing this antigen were retained for this analysis.

### 4.2. Single-Cell RNA Analysis

For the analysis of the scRNA datasets, the Seurat v5 pipeline [68] (https://satijalab.org/seurat/articles/integration_introduction, accessed on 8 January 2024) was applied, while each dataset was first analysed separately.

The main analysis began with the creation of a Seurat object for each sample, requiring a minimum of 3 cells and 200 features. All Seurat objects were subsequently merged. Then, normalisation of the count data, feature selection, and data scaling were followed. Next, Principal Component Analysis (PCA) was performed, followed by neighbour finding and clustering on the unintegrated dataset. To visualise the results, the Uniform Manifold Approximation and Projection (UMAP) method was applied. For the integration of the datasets, an anchor-based canonical correlation analysis (CCA) [69] approach was selected. CCA identifies linear combinations of features across multiple datasets that exhibit maximal correlation, making it particularly effective for uncovering shared correlation structures between datasets. Notably, its application outperformed traditional linear batch correction techniques such as ComBat and limma, yielding superior results in preserving biological signals while minimising batch effects. Furthermore, CCA has been widely utilised in multimodal genomic analyses of bulk samples, where it has proven valuable in revealing relationships between gene expression levels and DNA copy number variations within the same sample set [69].

After integration, the RNA layers of all samples were merged, followed by neighbour finding and clustering. Finally, given that all cells were derived from CD138^+^ PCs, all clusters were combined into a single group named “Plasma Cells.”

To generate the final integrated dataset for our downstream analyses, we first merged outputs from the previous analysis [70]. Then, we reapplied all preprocessing steps originally performed on the individual datasets and used CCA integration to correct the batch effect between the two datasets. Finally, DEA was performed on the integrated dataset using the Wilcoxon test. For each stage comparison, *p*-values, adjusted *p*-values, and log_2_FC values were obtained for each gene. Finally, lists of DEGs for all stages were generated based on a p_adj_ ≤ 0.05 threshold and are available in the Supplementary File S8.

### 4.3. Regulatory Network Construction and Analysis

For developing stage-specific regulatory networks, DEGs with an absolute log_2_FC ≥ 1, along with those present in MMCG set, were used. Those genes were used as queries to the complete Signor v3.0 database (https://signor.uniroma2.it/about/, accessed on 23 May 2025, downloaded: 23 May 2025) [54] after filtering the database based on the information source (Uniprot) and the organism (9606, Humans). Manual inspection of the data revealed duplicate gene–gene relationships due to multiple entries in SIGNOR. Since SIGNOR is manually curated from individual publications, the same interaction may appear more than once, each time linked to a different source. To address this, and because each relationship was assigned a single SIGNOR score without accounting for the number of supporting entries, we retained unique gene–gene relationships.

To analyse those networks, we used the igraph package in R [56] to identify the degree (in and out) of each node, and the Influential package in R, which calculates the Integrated Value of Influence (IVI) for each node. Because our networks were treated as directed, IVI scores (In, Out, All) were calculated. IVI score integrates the most significant network centrality measures to synergise their effects and simultaneously remove their biases to identify the most essential regulatory molecules in a network [55]. All loops representing self-regulatory interactions were removed prior to analysis, and all networks were visualised using the tidygraph (version: 1.3.1) [71] and ggraph (version: 2.2.2) [72] packages in R and Cytoscape (version: 3.10.2).

Filtering/ranking of top genes: To enrich our list and since degree is a component of the IVI metrics, genes with IVI_in and IVI_out scores ≥ 25 were retained for downstream analysis. This threshold aimed to identify genes with significant regulatory impact, either as major influencers or key targets within the network, warranting further investigation.

## 5. Conclusions

By employing a network-based approach in combination with single-cell analytics, we aimed to uncover key genes and molecular mechanisms involved in MM and its precursor stages, MGUS and SMM. Our analysis identified several influential genes—*NR3C1*, *HDAC1*, *GSK3B*, *CDKN1A*, *RELA*, and *PCK2*—which emerged as central nodes across all disease stages. Further investigation of influential genes not previously included in the MMCG set highlighted *GSK3B*, *CDKN1A*, and *PCK2* as being consistently present in all three stages. In addition, 24 stage-specific genes were identified: twelve specific to MGUS (*SGK3*, *MACF1*, *CAPN1*, *CAPN2*, *ILK*, *LAT*, *PPM1A*, *PTEN*, *PTPRC*, *ROCK1*, *BAX*, *VAV1*), six for SMM (*EIF2B5*, *ERN1*, *NFE2L1*, *PSIP1*, *SFPQ*, *ZC3HAV1*), and six for MM (*CDK5*, *PRKCD*, *PRKDC*, *HSPA5*, *MDM2*, *NFATC2*). Among these, *GSK3B*, *PCK2*, *MACF1*, *CAPN1*, *CAPN2*, *PTPRC*, *VAV1*, *EIF2B5*, *PSIP1*, *SFPQ*, *ZC3HAV1*, *PRKCD*, and *MDM2* are promising for further investigation based on our findings.

As a next step, these findings provide a strong rationale to functionally investigate *GSK3B* and *PCK2*, along with the stage-specific genes, in cell lines and patient samples. Such studies could identify therapeutic vulnerabilities and guide the development of drug strategies, either as monotherapies or in combination with established MM treatments, such as bortezomib, with the ultimate goal of halting disease progression from MGUS and SMM to active MM.

## Figures and Tables

**Table 1:** Top 10 genes according to the Integrated Value of Influence (IVI) Score (ALL).

MGUS						
Gene	Degree (IN)	Degree (OUT)	Degree (ALL)	IVI Score (IN)	IVI Score (OUT)	IVI Score (ALL)
*GSK3B*	6	23	29	52.1	74.4	100.0
*AKT2*	1	6	7	4.1	100.0	24.0
*PTEN*	5	2	7	46.9	11.1	22.6
*ILK*	1	4	5	12.1	56.8	20.4
*PRKCD*	0	15	15	1.0	1.4	14.6
*LCK*	1	8	9	6.0	37.3	12.9
*CTNNB1*	6	3	9	13.8	1.4	12.9
*BAX*	7	1	8	75.2	1.3	12.7
*ROCK1*	2	5	7	57.4	8.2	11.0
*JUN*	4	1	5	100.0	3.2	10.0
**SMM**						
**Gene**	**Degree (IN)**	**Degree (OUT)**	**Degree (ALL)**	**IVI Score (IN)**	**IVI Score (OUT)**	**IVI Score (ALL)**
*GSK3B*	4	11	15	40.0	100.0	100.0
*HDAC3*	1	1	2	59.6	20.8	10.7
*NR3C1*	1	1	2	55.2	11.4	8.6
*NFKBIA*	3	1	4	4.7	8.8	6.8
*HDAC1*	1	2	3	25.1	14.2	6.8
*AKT2*	0	2	2	2.3	9.7	6.6
*RELA*	2	1	3	100.0	4.9	6.0
*HSPA5*	2	1	3	23.8	8.8	5.4
*CDKN1A*	3	0	3	26.9	4.7	4.1
*SFPQ*	1	1	2	46.4	4.8	4.0
**MM**						
**Gene**	**Degree (IN)**	**Degree (OUT)**	**Degree (ALL)**	**IVI Score (IN)**	**IVI Score (OUT)**	**IVI Score (ALL)**
*GSK3B*	2	16	18	20.5	75.2	100.0
*ATM*	2	6	8	100.0	100.0	76.3
*CDK5*	1	3	4	18.6	44.7	32.4
*PRKCD*	0	10	10	1.2	43.6	21.1
*MYC*	1	9	10	22.8	5.9	19.1
*MDM2*	1	7	8	44.3	3.8	15.8
*HSPA5*	2	2	4	80.1	6.1	13.3
*BRAF*	2	2	4	54.4	17.3	12.5
*PRKDC*	1	3	4	39.6	9.4	9.9
*HDAC1*	2	2	4	28.4	4.2	9.8

**Table 2:** Highlighted genes by our analysis.

MGUS		SMM		MM	
Gene	MMCG	Gene	MMCG	Gene	MMCG
*AKT2*	YES	*BICD1*	YES	*ATM*	YES
*BBC3*	YES	*CCND1*	YES	*BRAF*	YES
*BCL2L11*	YES	*CCND3*	YES	*HDAC1*	YES
*DUSP1*	YES	*HDAC1*	YES	*RELA*	YES
*JUN*	YES	*HDAC3*	YES	*GSK3B*	NO
*LCK*	YES	*MCL1*	YES	*CDKN1A*	NO
*LEF1*	YES	*NR3C1*	YES	*PCK2*	NO
*NR3C1*	YES	*RELA*	YES	*CDK5*	NO
*RELA*	YES	*GSK3B*	NO	*PRKCD*	NO
*GSK3B*	NO	*CDKN1A*	NO	*PRKDC*	NO
*CDKN1A*	NO	*PCK2*	NO	*HSPA5*	NO
*PCK2*	NO	*EIF2B5*	NO	*MDM2*	NO
*SGK3*	NO	*ERN1*	NO	*NFATC2*	NO
*MACF1*	NO	*NFE2L1*	NO		
*CAPN1*	NO	*PSIP1*	NO		
*CAPN2*	NO	*SFPQ*	NO		
*ILK*	NO	*ZC3HAV1*	NO		
*LAT*	NO				
*PPM1A*	NO				
*PTEN*	NO				
*PTPRC*	NO				
*ROCK1*	NO				
*BAX*	NO				
*VAV1*	NO				

**Table 3:** Genes highlighted by our analysis that were not part of the MMCG set, together with their corresponding IVI scores. All listed genes exhibit at least one IVI score (in, out, and all) greater than 25, which is the defined significance threshold. For each gene, the maximum IVI score is reported in the Max IVI score column, while the corresponding IVI value is highlighted in bold within the table.

Gene	Stage	In IVI	Out IVI	All IVI	Max IVI Score
*GSK3B*	MGUS	52.1	74.4	**100.0**	100.0
*SGK3*	MGUS	7.5	**97.5**	7.1	97.5
*PTPRC*	MGUS	4.7	**76.0**	6.4	76.0
*BAX*	MGUS	**75.2**	1.3	12.7	75.2
*MACF1*	MGUS	20.1	**60.8**	2.6	60.8
*CAPN1*	MGUS	1.0	**60.8**	1.4	60.8
*CAPN2*	MGUS	1.0	**60.8**	1.4	60.8
*ROCK1*	MGUS	**57.4**	8.2	11.0	57.4
*ILK*	MGUS	12.1	**56.8**	20.4	56.8
*VAV1*	MGUS	**55.5**	50.2	8.8	55.5
*PTEN*	MGUS	**46.9**	11.1	22.6	46.9
*PPM1A*	MGUS	**38.1**	6.4	4.9	38.1
*PCK2*	MGUS	**36.2**	1.0	1.4	36.2
*CDKN1A*	MGUS	**28.7**	1.0	7.1	28.7
*LAT*	MGUS	**26.2**	8.5	6.5	26.2
*GSK3B*	SMM	40.0	**100.0**	**100.0**	100.0
*SFPQ*	SMM	**46.4**	4.8	4.0	46.4
*EIF2B5*	SMM	**42.0**	4.7	1.6	42.0
*NFE2L1*	SMM	**42.0**	4.7	1.6	42.0
*ZC3HAV1*	SMM	**42.0**	4.7	1.6	42.0
*ERN1*	SMM	**39.7**	4.8	1.9	39.7
*PCK2*	SMM	**33.4**	4.7	1.1	33.4
*PSIP1*	SMM	**33.4**	4.7	1.1	33.4
*CDKN1A*	SMM	**26.9**	4.7	4.1	26.9
*GSK3B*	MM	20.5	75.2	**100.0**	100.0
*HSPA5*	MM	**80.1**	6.1	13.3	80.1
*CDK5*	MM	18.6	**44.7**	32.4	44.7
*MDM2*	MM	**44.3**	3.8	15.8	44.3
*PRKCD*	MM	1.2	**43.6**	21.1	43.6
*PRKDC*	MM	**39.6**	9.4	9.9	39.6
*CDKN1A*	MM	**37.8**	1.0	5.7	37.8
*PCK2*	MM	**36.2**	1.0	1.6	36.2
*NFATC2*	MM	**26.6**	1.1	5.2	26.6

**Table 4:** Summary of CD138^+^ scRNA datasets samples.

Disease	GSE145977	EGAD00001009648
HEALTHY	9	1
MGUS	6	6
SMM	12	7
MM	8	5

## Data Availability

The datasets analysed in the article are accessible from the Gene Expression Omnibus (GEO) database (https://www.ncbi.nlm.nih.gov/geo/, accessed: 08 January 2024) and the European Genome-Phenotype Archive (EGA) (https://ega-archive.org/, accessed: 08 January 2024). The source codes are available upon request.

## Supplementary Materials

The following supporting information can be downloaded at https://www.mdpi.com/article/10.3390/ijms27020793/s1.

## References

[1] Xu Z., Yu J., Chen Y. (2024). Hub genes and associated drugs for multiple myeloma with 1q21+: Identified by bioinformatic analysis. Hematology.

[2] Dong X., Lu G., Su X., Liu J., Chen X., Tian Y., Chang Y., Wang L., Wang W., Zhou J. (2021). Identification of key miRNA signature and pathways involved in multiple myeloma by integrated bioinformatics analysis. Hematology.

[3] Borad A., Saeed H., Toscani M., Barone J., Weber P. (2020). Age demographics of subjects enrolled in interventional phase 3 multiple myeloma clinical trials. J. Oncol. Pharm. Pract..

[4] Peng Y., Wu D., Li F., Zhang P., Feng Y., He A. (2020). Identification of key biomarkers associated with cell adhesion in multiple myeloma by integrated bioinformatics analysis. Cancer Cell Int..

[5] Zuern K., Hielscher T., Werly A., Breitkreutz I., Sauer S., Raab M.S., Müller-Tidow C., Goldschmidt H., Mai E.K. (2024). Longitudinal assessment of established risk stratification models in patients with monoclonal gammopathy of undetermined significance. Blood Cancer J..

[6] Rajkumar S.V., Mateos M.-V., Schaeffer M., Lin X., Bathija S., Gupta-Werner N., Lam A., Carson R., Dennis R., Kaila S. (2024). Real-world characteristics and outcomes of patients with high-risk and non-high-risk smoldering multiple myeloma using the Flatiron Health database. Blood Cancer J..

[7] Shi J., Lu Y., Wei W., Ma G., Li C., Li L., Wang Y., Wang Y., Xu R., Cui S. (2025). Ferroptosis: A novel pharmacological mechanism against multiple myeloma. Front. Pharmacol..

[8] Ho M., Patel A., Goh C.Y., Moscvin M., Zhang L., Bianchi G. (2020). Changing paradigms in diagnosis and treatment of monoclonal gammopathy of undetermined significance (MGUS) and smoldering multiple myeloma (SMM). Leukemia.

[9] Savva K., Bourdakou M.M., Stellas D., Zoidakis J., Spyrou G.M. (2025). Computational Drug Repurposing Across the Multiple Myeloma Spectrum: From MGUS to MM. Cancers.

[10] Saadoune C., Nouadi B., Hamdaoui H., Chegdani F., Bennis F. (2022). Multiple Myeloma: Bioinformatic Analysis for Identification of Key Genes and Pathways. Bioinform. Biol. Insights.

[11] Riccardi F., Tangredi C., Dal Bo M., Toffoli G. (2024). Targeted therapy for multiple myeloma: An overview on CD138-based strategies. Front. Oncol..

[12] Wang Y., Zhang W., Li T., Liu M., Gao M., Li X., Chen Y., Song Y., Li W., Du C. (2024). Identification of potential immune-related mechanisms related to the development of multiple myeloma. Chin. Med. J..

[13] Cardona-Benavides I.J., de Ramón C., Gutiérrez N.C. (2021). Genetic Abnormalities in Multiple Myeloma: Prognostic and Therapeutic Implications. Cells.

[14] Castaneda O., Baz R. (2019). Multiple Myeloma Genomics—A Concise Review. Acta Med. Acad..

[15] van Nieuwenhuijzen N., Spaan I., Raymakers R., Peperzak V. (2018). From MGUS to Multiple Myeloma, a Paradigm for Clonal Evolution of Premalignant Cells. Cancer Res..

[16] Alaterre E., Ovejero S., Herviou L., de Boussac H., Papadopoulos G., Kulis M., Boireau S., Robert N., Requirand G., Bruyer A. (2022). Comprehensive characterization of the epigenetic landscape in Multiple Myeloma. Theranostics.

[17] Manier S., Salem K.Z., Park J., Landau D.A., Getz G., Ghobrial I.M. (2017). Genomic complexity of multiple myeloma and its clinical implications. Nat. Rev. Clin. Oncol..

[18] Boyle E.M., Deshpande S., Tytarenko R., Ashby C., Wang Y., Bauer M.A., Johnson S.K., Wardell C.P., Thanendrarajan S., Zangari M. (2021). The molecular make up of smoldering myeloma highlights the evolutionary pathways leading to multiple myeloma. Nat. Commun..

[19] Mikulasova A., Wardell C.P., Murison A., Boyle E.M., Jackson G.H., Smetana J., Kufova Z., Pour L., Sandecka V., Almasi M. (2017). The spectrum of somatic mutations in monoclonal gammopathy of undetermined significance indicates a less complex genomic landscape than that in multiple myeloma. Haematologica.

[20] Hultcrantz M., Yellapantula V., Rustad E.H. (2020). Genomic profiling of multiple myeloma: New insights and modern technologies. Best Pract. Res. Clin. Haematol..

[21] Rajan A.M., Rajkumar S.V. (2015). Interpretation of cytogenetic results in multiple myeloma for clinical practice. Blood Cancer J..

[22] Manier S., Kawano Y., Bianchi G., Roccaro A.M., Ghobrial I.M. (2016). Cell autonomous and microenvironmental regulation of tumor progression in precursor states of multiple myeloma. Curr. Opin. Hematol..

[23] Wu D., Zhang P., Li F., Shen Y., Chen H., Feng Y., He A., Wang F. (2020). CD138- multiple myeloma cells express high level of CHK1 which correlated to overall survival in MM patient. Aging.

[24] Sayers E.W., Beck J., Bolton E.E., Brister J.R., Chan J., Connor R., Feldgarden M., Fine A.M., Funk K., Hoffman J. (2025). Database resources of the National Center for Biotechnology Information in 2025. Nucleic Acids Res..

[25] Bai H., Chen B. (2020). A 5-Gene Stemness Score for Rapid Determination of Risk in Multiple Myeloma. Onco Targets Ther..

[26] Zeng J., Luo Q., Wang X., Xie W., Dong S., Fu H., Wei Y., Liu T. (2023). Network pharmacology- and molecular docking-based investigation of the therapeutic potential and mechanism of daucosterol against multiple myeloma. Transl. Cancer Res..

[27] Golovina E., Kokavec J., Kazantsev D., Yurikova O., Bajecny M., Savvulidi F.G., Simersky R., Lenobel R., Tost J., Herynek V. (2025). Deficiency of miR-155 in Leukemic B-Cells Results in Cell Cycle Arrest and Deregulation of MIR155HG/TP53INP1/CDKN1A/CCND1 network. Arch. Med. Res..

[28] Katiyar A., Kaur G., Rani L., Jena L., Singh H., Kumar L., Sharma A., Kaur P., Gupta R. (2021). Genome-wide identification of potential biomarkers in multiple myeloma using meta-analysis of mRNA and miRNA expression data. Sci. Rep..

[29] Tuerxun N., Wang J., Qin Y.-T., Zhao F., Wang H., Qu J.-H., Uddin M.N., Hao J.-P. (2022). Identification of key genes and miRNA-mRNA regulatory networks associated with bone marrow immune microenvironment regulations in multiple myeloma by integrative bioinformatics analysis. Hematology.

[30] Papanagnou E.-D., Terpos E., Kastritis E., Papassideri I.S., Tsitsilonis O.E., Dimopoulos M.A., Trougakos I.P. (2018). Molecular responses to therapeutic proteasome inhibitors in multiple myeloma patients are donor-, cell type- and drug-dependent. Oncotarget.

[31] Wang F., Gao Y., Chen Y., Li P., Zeng Y., Chen Y., Yin Y., Jia Y., Wang Y. (2025). Development of a mitochondria-related gene signature for prognostic assessment in diffuse large B cell lymphoma. Front. Oncol..

[32] Uhlén M., Fagerberg L., Hallström B.M., Lindskog C., Oksvold P., Mardinoglu A., Sivertsson Å., Kampf C., Sjöstedt E., Asplund A. (2015). Proteomics. Tissue-based map of the human proteome. Science.

[33] Hausmann S., Brandt E., Köchel C., Einsele H., Bargou R.C., Seggewiss-Bernhardt R., Stühmer T. (2015). Loss of serum and glucocorticoid-regulated kinase 3 (SGK3) does not affect proliferation and survival of multiple myeloma cell lines. PLoS ONE.

[34] Miao Z., Ali A., Hu L., Zhao F., Yin C., Chen C., Yang T., Qian A. (2017). Microtubule actin cross-linking factor 1, a novel potential target in cancer. Cancer Sci..

[35] Shapovalov I., Rimal P., Poudel P., Lewtas V., Bell M., Panday S.K., Laight B.J., Harper D., Grieve S., Baillie G.S. (2025). Quantification and structure-function analysis of calpain-1 and calpain-2 protease subunit interactions. J. Biol. Chem..

[36] Zhan F., Tian E., Bumm K., Smith R., Barlogie B., Shaughnessy J. (2003). Gene expression profiling of human plasma cell differentiation and classification of multiple myeloma based on similarities to distinct stages of late-stage B-cell development. Blood.

[37] Steinbrunn T., Siegmund D., Andrulis M., Grella E., Kortüm M., Einsele H., Wajant H., Bargou R.C., Stühmer T. (2012). Integrin-linked kinase is dispensable for multiple myeloma cell survival. Leuk. Res..

[38] Zhao W., Zhang X., Zang L., Zhao P., Chen Y., Wang X. (2018). ILK promotes angiogenic activity of mesenchymal stem cells in multiple myeloma. Oncol. Lett..

[39] Alimohammadi M., Rahimzadeh P., Khorrami R., Bonyadi M., Daneshi S., Nabavi N., Raesi R., Farani M.R., Dehkhoda F., Taheriazam A. (2024). A comprehensive review of the PTEN/PI3K/Akt axis in multiple myeloma: From molecular interactions to potential therapeutic targets. Pathol. Res. Pract..

[40] Papadimitriou M.-A., Soureas K., Papanota A.-M., Tsiakanikas P., Adamopoulos P.G., Ntanasis-Stathopoulos I., Malandrakis P., Gavriatopoulou M., Sideris D.C., Kastritis E. (2023). miRNA-seq identification and clinical validation of CD138+ and circulating miR-25 in treatment response of multiple myeloma. J. Transl. Med..

[41] Kamal M., Shishido S.N., Mason J., Patel K., Manasanch E.E., Orlowski R.Z., Kuhn P. (2025). Single-cell proteomic analysis reveals Multiple Myeloma heterogeneity and the dynamics of the tumor immune microenvironment in precursor and advanced states. Neoplasia.

[42] López-Corral L., Corchete L.A., Sarasquete M.E., Mateos M.V., García-Sanz R., Fermiñán E., Lahuerta J.-J., Bladé J., Oriol A., Teruel A.I. (2014). Transcriptome analysis reveals molecular profiles associated with evolving steps of monoclonal gammopathies. Haematologica.

[43] Kharabi Masouleh B., Geng H., Hurtz C., Chan L.N., Logan A.C., Chang M.S., Huang C., Swaminathan S., Sun H., Paietta E. (2014). Mechanistic rationale for targeting the unfolded protein response in pre-B acute lymphoblastic leukemia. Proc. Natl. Acad. Sci. USA.

[44] Sedlacek J. (2025). Impact of proteostasis workload on sensitivity to proteasome inhibitors in multiple myeloma. Clin. Exp. Med..

[45] Ortiz-Hernandez G.L., Sanchez-Hernandez E.S., Ochoa P.T., Casiano C.A. (2024). The Emerging Roles of the Stress Epigenetic Reader LEDGF/p75 in Cancer Biology and Therapy Resistance: Mechanisms and Targeting Opportunities. Cancers.

[46] Laurenzi T., Palazzolo L., Taiana E., Saporiti S., Ben Mariem O., Guerrini U., Neri A., Eberini I. (2022). Molecular Modelling of NONO and SFPQ Dimerization Process and RNA Recognition Mechanism. Int. J. Mol. Sci..

[47] Huang W., Hua H., Xiao G., Yang X., Yang Q., Jin L. (2021). ZC3HAV1 promotes the proliferation and metastasis via regulating KRAS in pancreatic cancer. Aging.

[48] Tang H., Xu L., Cen X., Yang L., Feng J., Li G., Zhu H., Gao S., Yu Y., Zhao Y. (2020). CDK5 inhibition in vitro and in vivo induces cell death in myeloma and overcomes the obstacle of bortezomib resistance. Int. J. Mol. Med..

[49] Liu J., Du F., Chen C., Li D., Chen Y., Xiao X., Hou X. (2020). CircRNA ITCH increases bortezomib sensitivity through regulating the miR-615-3p/PRKCD axis in multiple myeloma. Life Sci..

[50] Ortiz-Estévez M., Towfic F., Flynt E., Stong N., Jang I.S., Wang K., Trotter M.W.B., Thakurta A. (2021). Integrative multi-omics identifies high risk multiple myeloma subgroup associated with significant DNA loss and dysregulated DNA repair and cell cycle pathways. BMC Med. Genom..

[51] Jovanović K.K., Escure G., Demonchy J., Willaume A., Van de Wyngaert Z., Farhat M., Chauvet P., Facon T., Quesnel B., Manier S. (2018). Deregulation and Targeting of TP53 Pathway in Multiple Myeloma. Front. Oncol..

[52] Schütt J., Nägler T., Schenk T., Brioli A. (2021). Investigating the Interplay between Myeloma Cells and Bone Marrow Stromal Cells in the Development of Drug Resistance: Dissecting the Role of Epigenetic Modifications. Cancers.

[53] Yang Q., Li K., Li X., Liu J. (2020). Identification of Key Genes and Pathways in Myeloma side population cells by Bioinformatics Analysis. Int. J. Med. Sci..

[54] Lo Surdo P., Iannuccelli M., Contino S., Castagnoli L., Licata L., Cesareni G., Perfetto L. (2023). SIGNOR 3.0, the SIGnaling network open resource 3.0: 2022 update. Nucleic Acids Res..

[55] Salavaty A., Ramialison M., Currie P.D. (2020). Integrated Value of Influence: An Integrative Method for the Identification of the Most Influential Nodes within Networks. Patterns.

[56] Csárdi G., Nepusz T., Müller K., Horvát S., Traag V., Zanini F., Noom D. (2024). Igraph for R: R Interface of the Igraph Library for Graph Theory and Network Analysis.

[57] Lin Y., Mehta S., Küçük-McGinty H., Turner J.P., Vidovic D., Forlin M., Koleti A., Nguyen D.-T., Jensen L.J., Guha R. (2017). Drug target ontology to classify and integrate drug discovery data. J. Biomed. Semant..

[58] Moreno P., Fexova S., George N., Manning J.R., Miao Z., Mohammed S., Muñoz-Pomer A., Fullgrabe A., Bi Y., Bush N. (2022). Expression Atlas update: Gene and protein expression in multiple species. Nucleic Acids Res..

[59] Rappaport N., Twik M., Plaschkes I., Nudel R., Iny Stein T., Levitt J., Gershoni M., Morrey C.P., Safran M., Lancet D. (2017). MalaCards: An amalgamated human disease compendium with diverse clinical and genetic annotation and structured search. Nucleic Acids Res..

[60] Huang H.-Y., Lin Y.-C.-D., Li J., Huang K.-Y., Shrestha S., Hong H.-C., Tang Y., Chen Y.-G., Jin C.-N., Yu Y. (2020). miRTarBase 2020: Updates to the experimentally validated microRNA-target interaction database. Nucleic Acids Res..

[61] Sticht C., De La Torre C., Parveen A., Gretz N. (2018). miRWalk: An online resource for prediction of microRNA binding sites. PLoS ONE.

[62] Cui C., Zhong B., Fan R., Cui Q. (2024). HMDD v4.0: A database for experimentally supported human microRNA-disease associations. Nucleic Acids Res..

[63] Avram S., Wilson T.B., Curpan R., Halip L., Borota A., Bora A., Bologa C.G., Holmes J., Knockel J., Yang J.J. (2023). DrugCentral 2023 extends human clinical data and integrates veterinary drugs. Nucleic Acids Res..

[64] Knox C., Wilson M., Klinger C.M., Franklin M., Oler E., Wilson A., Pon A., Cox J., Chin N.E.L., Strawbridge S.A. (2024). DrugBank 6.0: The DrugBank Knowledgebase for 2024. Nucleic Acids Res..

[65] Whirl-Carrillo M., Huddart R., Gong L., Sangkuhl K., Thorn C.F., Whaley R., Klein T.E. (2021). An Evidence-Based Framework for Evaluating Pharmacogenomics Knowledge for Personalized Medicine. Clin. Pharmacol. Ther..

[66] Edgar R., Domrachev M., Lash A.E. (2002). Gene Expression Omnibus: NCBI gene expression and hybridization array data repository. Nucleic Acids Res..

[67] Dang M., Wang R., Lee H.C., Patel K.K., Becnel M.R., Han G., Thomas S.K., Hao D., Chu Y., Weber D.M. (2023). Single cell clonotypic and transcriptional evolution of multiple myeloma precursor disease. Cancer Cell.

[68] Hao Y., Stuart T., Kowalski M.H., Choudhary S., Hoffman P., Hartman A., Srivastava A., Molla G., Madad S., Fernandez-Granda C. (2023). Dictionary learning for integrative, multimodal and scalable single-cell analysis. Nat. Biotechnol..

[69] Butler A., Hoffman P., Smibert P., Papalexi E., Satija R. (2018). Integrating single-cell transcriptomic data across different conditions, technologies, and species. Nat. Biotechnol..

[70] Alexander P., Hugo T., Simon M., Vladimir K., Tallulah A., Jennifer W., Davis M., Maren B., Jimmy L., Krzysztof P. Analysis of Single Cell RNA-seq Data. https://www.singlecellcourse.org/scrna-seq-dataset-integration.html.

[71] Pedersen T.L. (2025). tidygraph: A Tidy API for Graph Manipulation. https://tidygraph.data-imaginist.com/.

[72] Pedersen T.L. (2025). ggraph: An Implementation of Grammar of Graphics for Graphs and Networks. https://ggraph.data-imaginist.com/.

