# Supplementary material for: Integrative Network Analysis of Single-Cell RNA Findings and a Priori Knowledge Highlights Gene Regulators in Multiple Myeloma Progression
Source: Int J Mol Sci. 2026 Jan 13;27(2):793. doi: 10.3390/ijms27020793 (PMC12840655; doi:10.3390/ijms27020793)
Supplement: Supplementary file 1 [file ijms-27-00793-s001.zip › SFile1.pdf]

Supplementary Materials

Table S1: Summary of filtering criteria used for each database.

Database	Filtering Criteria
DISGENET	Validated associations
Expression Atlas	No filtering
Malacards	Evidence-based associations
DrugCentral	Species: Homo Sapiens
DrugBank	Pharmacological action
PharmGKB	No filtering
HMDD	No filtering
mirTarBase	Species: Homo Sapiens
miRWALK	Validated associations<br>Binding score 0.8
UniProt	Species: Homo Sapiens

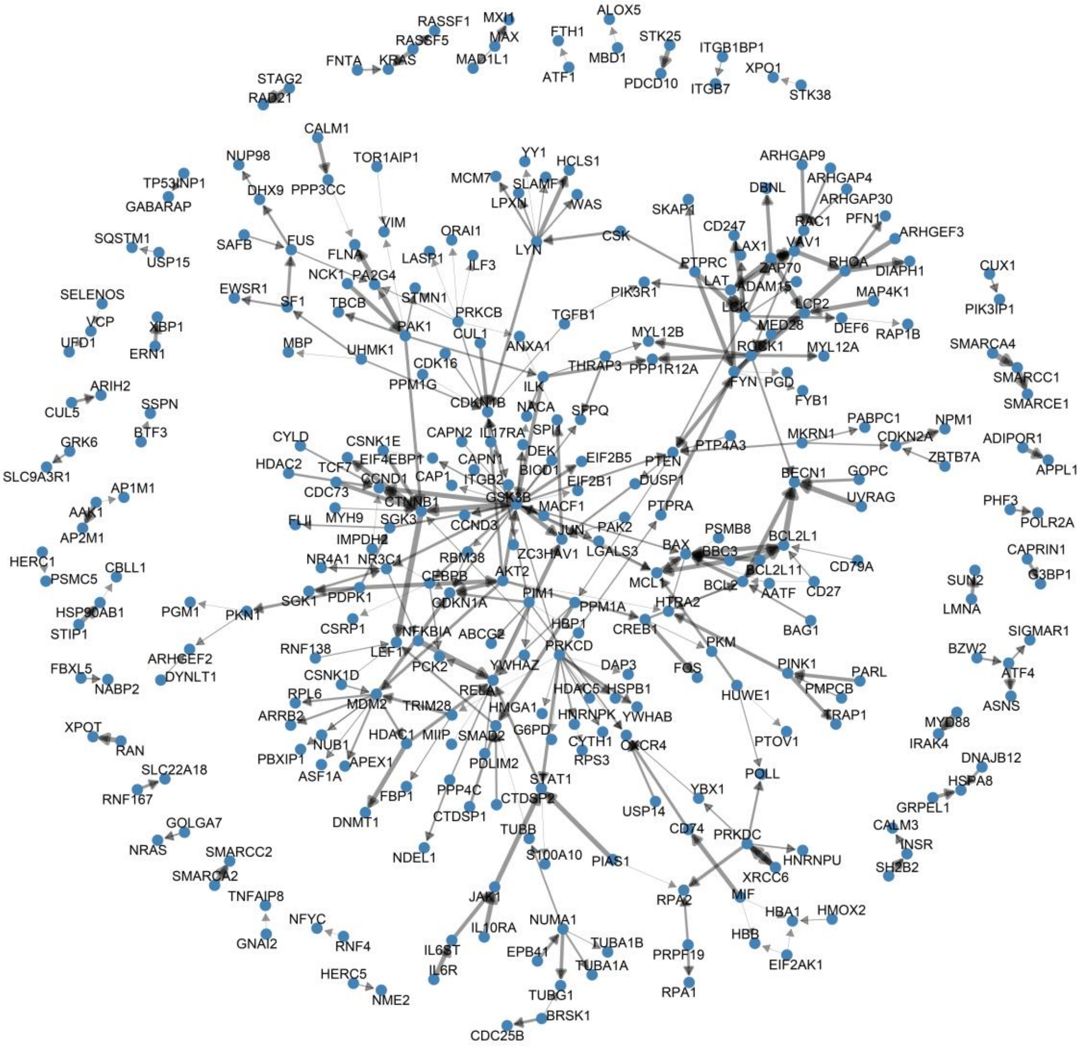

Figure S1: MGUS regulatory network. This network depicts the results of the queries from the SIGNOR database to create a stage-specific regulatory network for MGUS. Arrows depict the direction of the effect, while the edge width represents the Signor score.

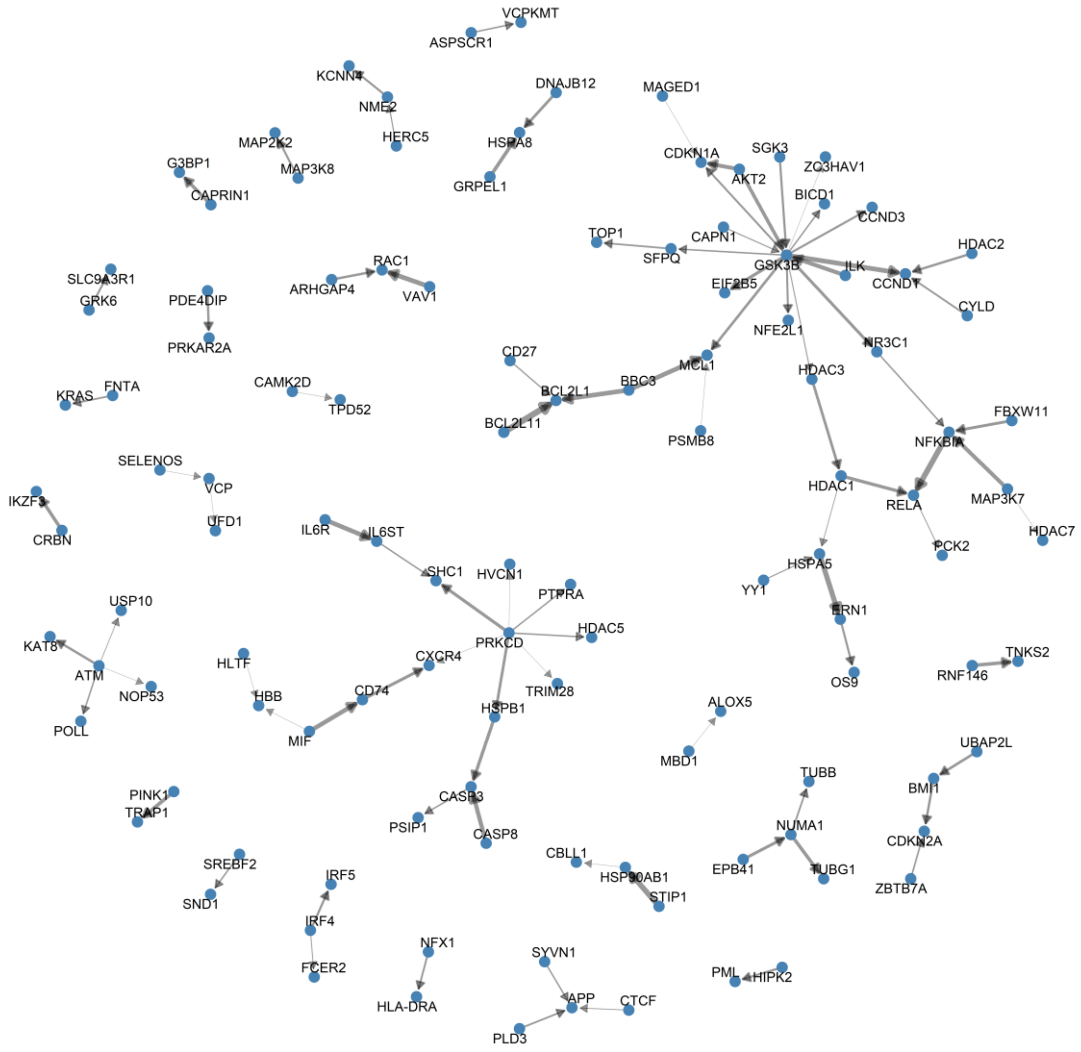

Figure S2: SMM regulatory network. This network depicts the results of the queries from the SIGNOR database to create a stage-specific regulatory network for SMM. Arrows depict the direction of the effect, while the edge width represents the Signor score.

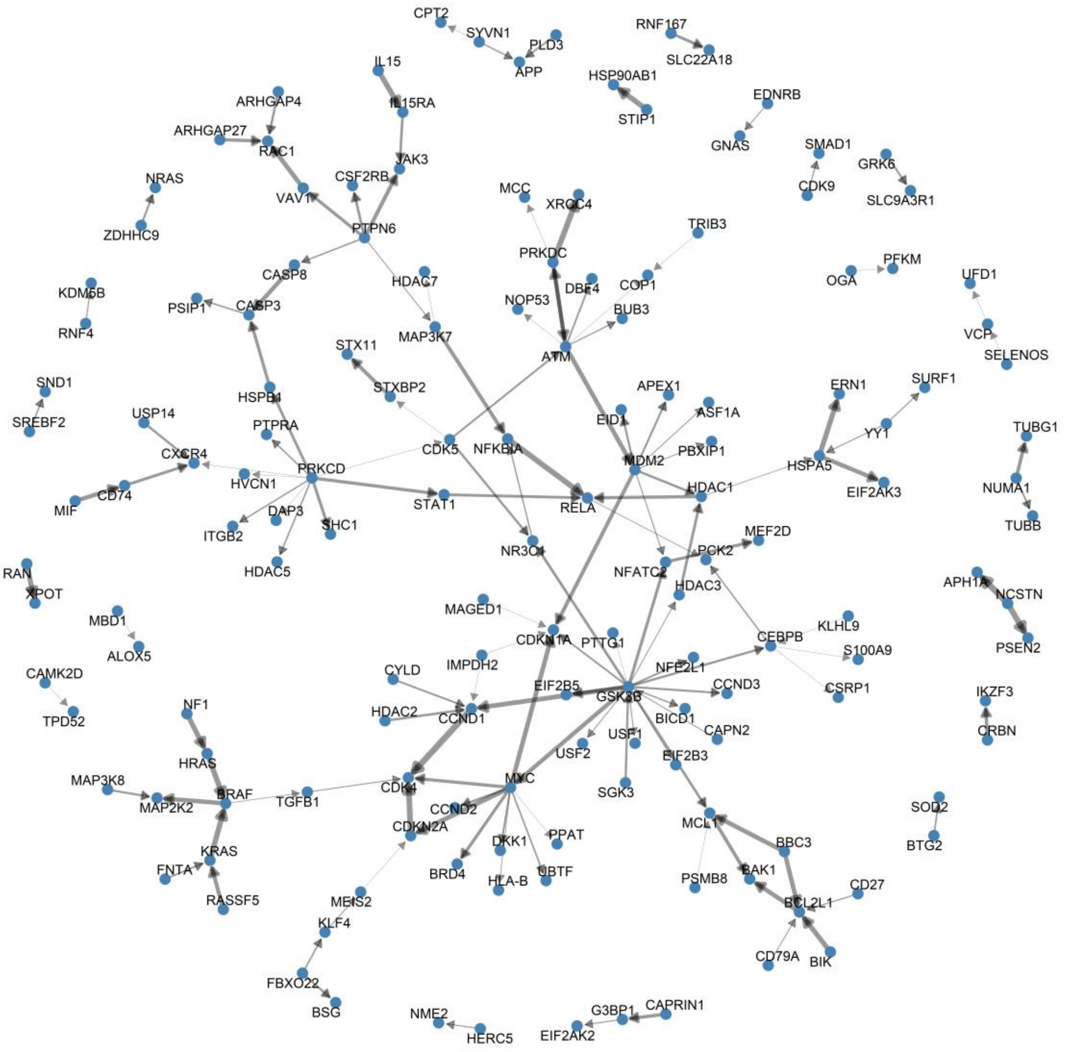

Figure S3: MM regulatory network. This network depicts the results of the queries from the SIGNOR database to create a stage-specific regulatory network for MM. Arrows depict the direction of the effect, while the edge width represents the SINGOR score.

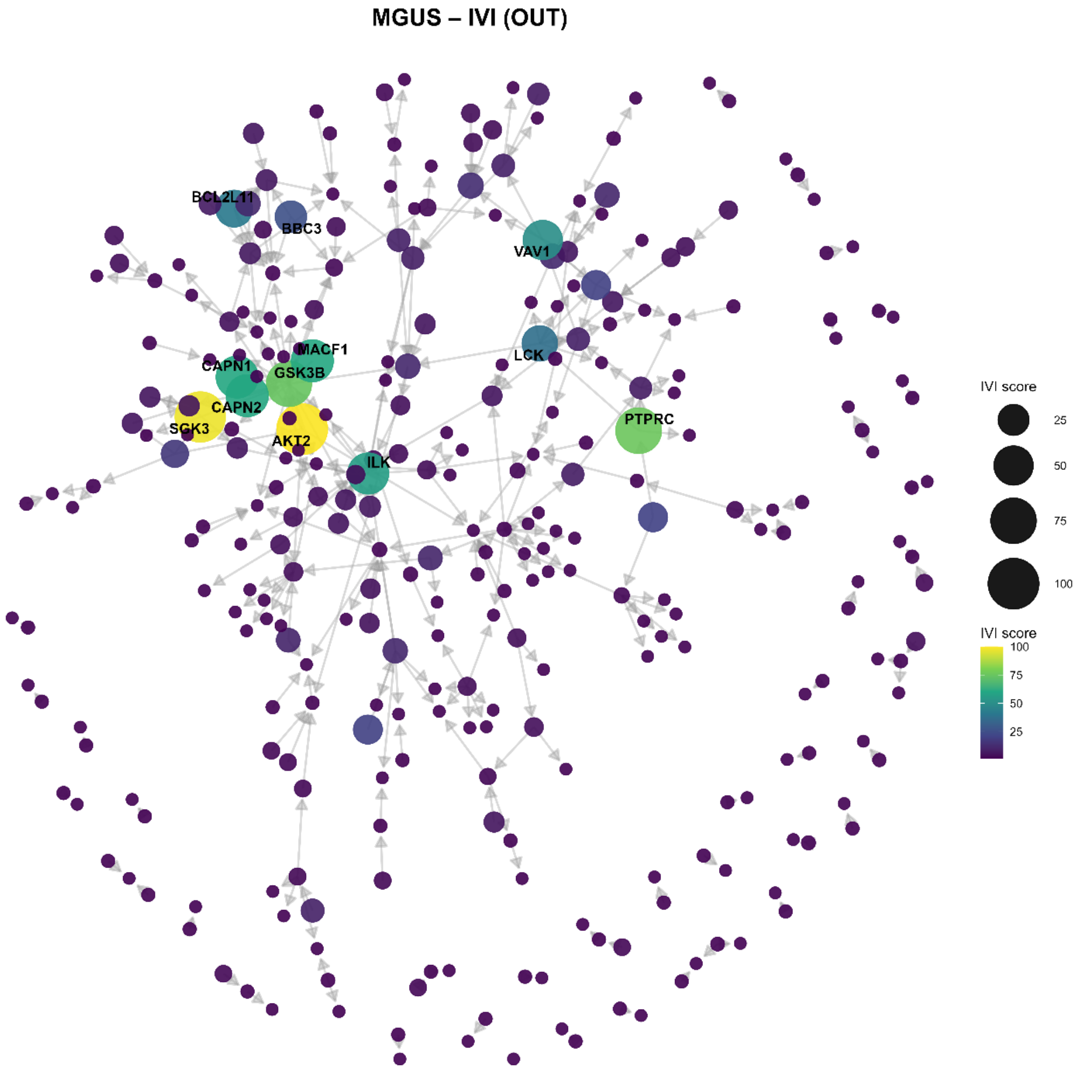

Figure S4: MGUS-Specific Network Comparison Based on IVI (OUT). This figure presents the MGUS-stage network with nodes sized and coloured according to their outgoing Influence Value Index (out_IVI), calculated using the Influential R package. Larger node sizes and more intense colours indicate stronger outgoing influence within the network. For clarity, only genes with out_IVI > 25 are labelled.

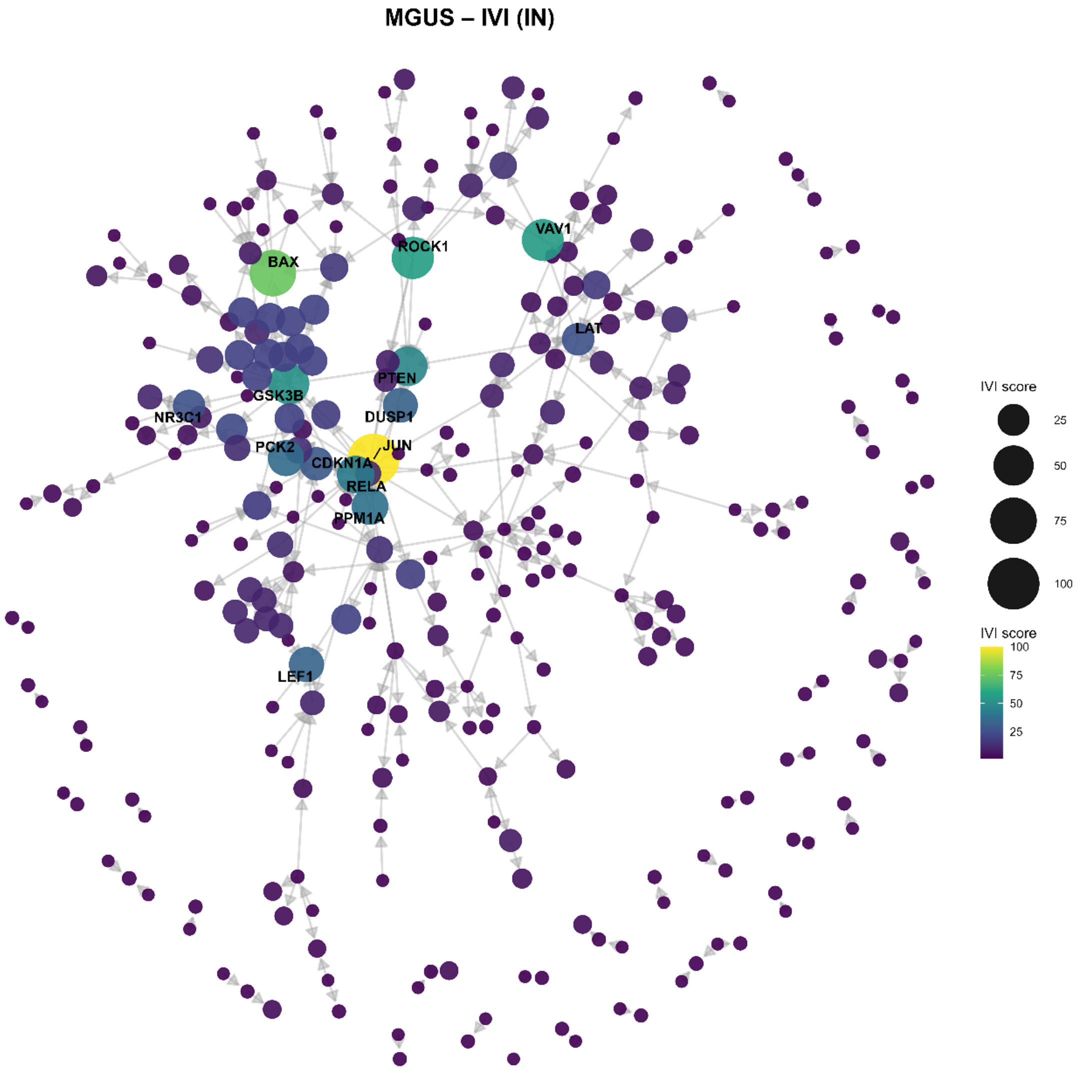

Figure S5: MGUS-Specific Network Comparison Based on IVI (IN). This figure presents the MGUS-stage network with nodes sized and coloured according to their outgoing Influence Value Index (in_IVI), calculated using the Influential R package. Larger node sizes and more intense colours indicate stronger outgoing influence within the network. For clarity, only genes with in_IVI > 25 are labelled.

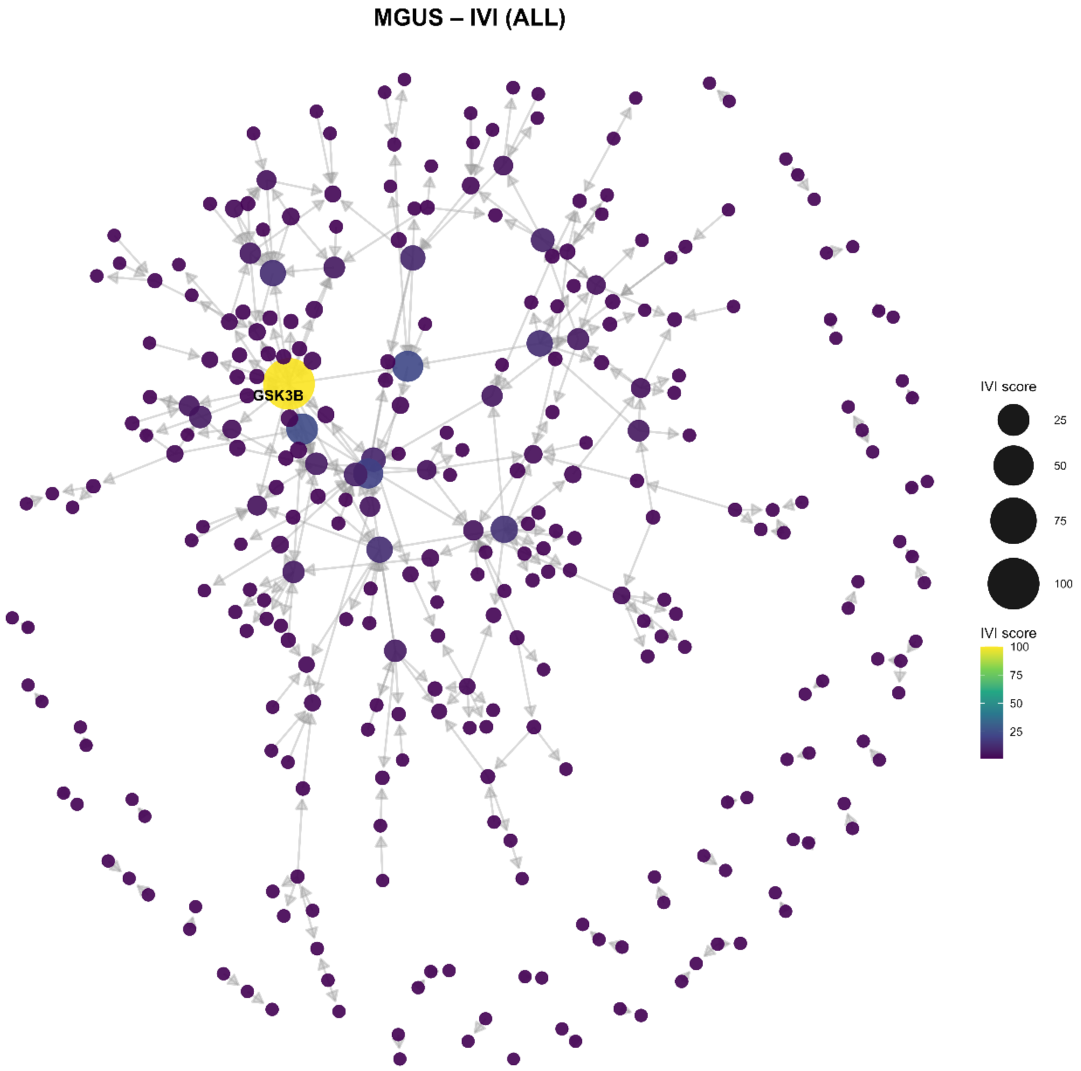

Figure S6: MGUS-Specific Network Comparison Based on IVI (ALL). This figure presents the MGUS-stage network with nodes sized and coloured according to their outgoing Influence Value Index (all_IVI), calculated using the Influential R package. Larger node sizes and more intense colours indicate stronger outgoing influence within the network. For clarity, only genes with all_IVI > 25 are labelled.

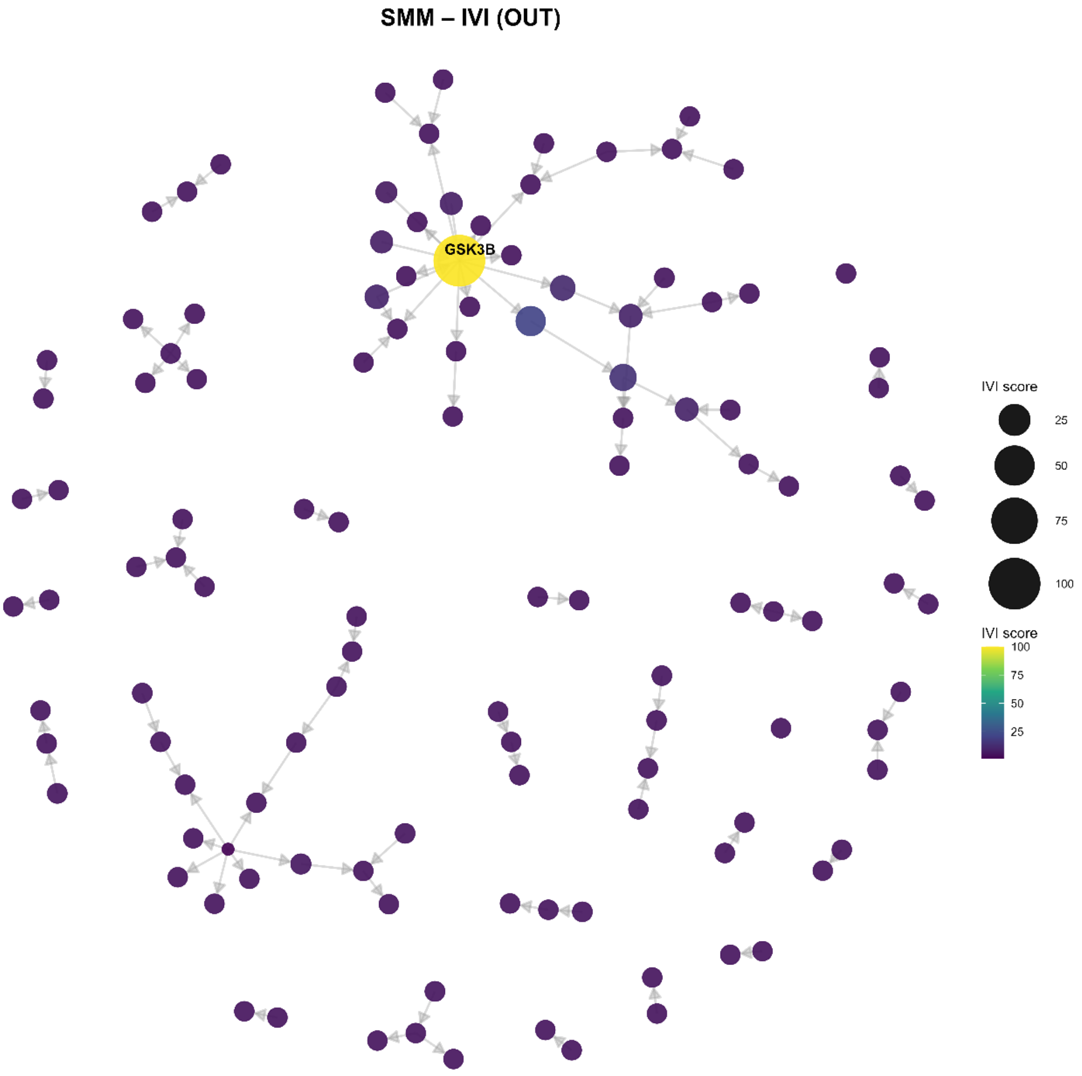

Figure S7: SMM-Specific Network Comparison Based on IVI (OUT). This figure presents the SMM-stage network with nodes sized and coloured according to their outgoing Influence Value Index (out_IVI), calculated using the Influential R package. Larger node sizes and more intense colours indicate stronger outgoing influence within the network. For clarity, only genes with out_IVI > 25 are labelled.

SMM – IVI (IN)

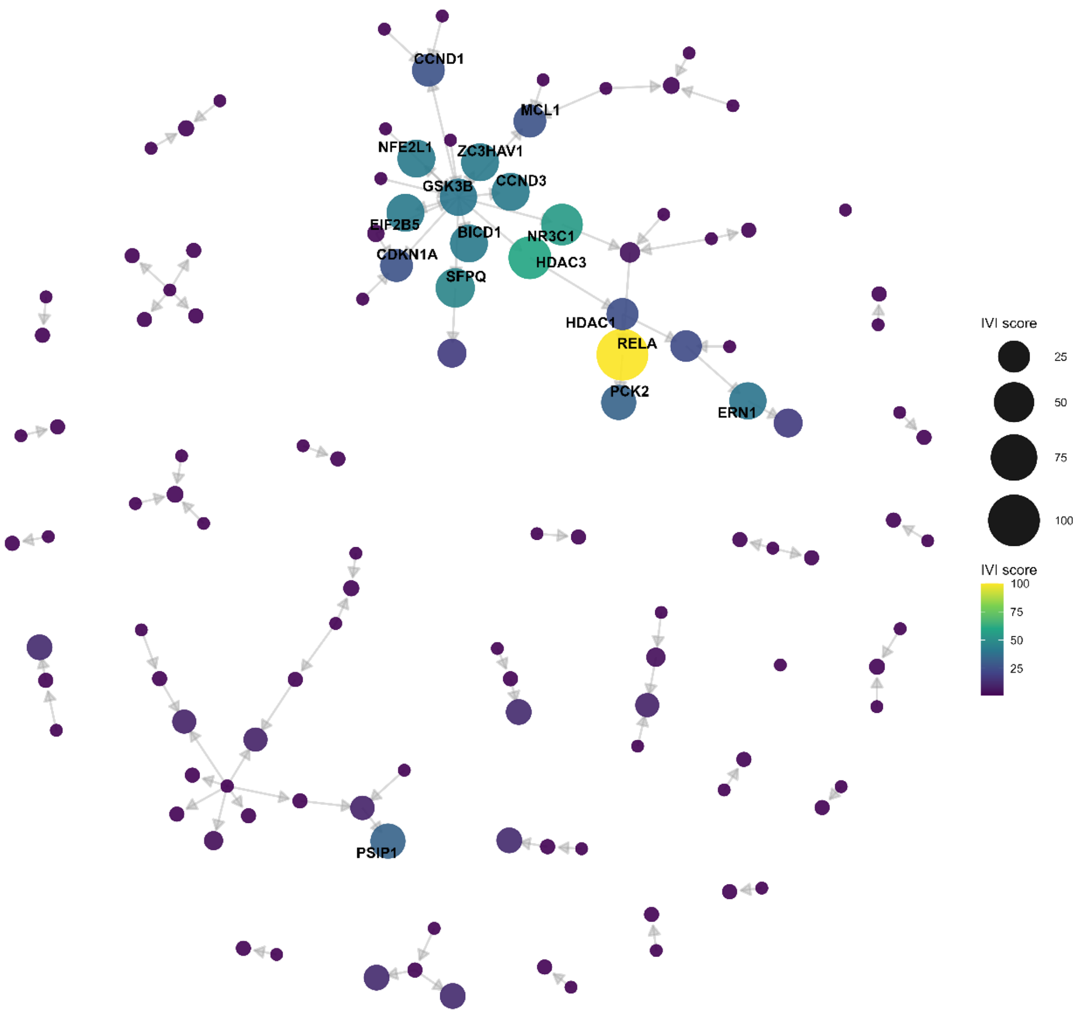

Figure S8: SMM-Specific Network Comparison Based on IVI (IN). This figure presents the SMM-stage network with nodes sized and coloured according to their outgoing Influence Value Index (in_IVI), calculated using the Influential R package. Larger node sizes and more intense colours indicate stronger outgoing influence within the network. For clarity, only genes with in_IVI > 25 are labelled.

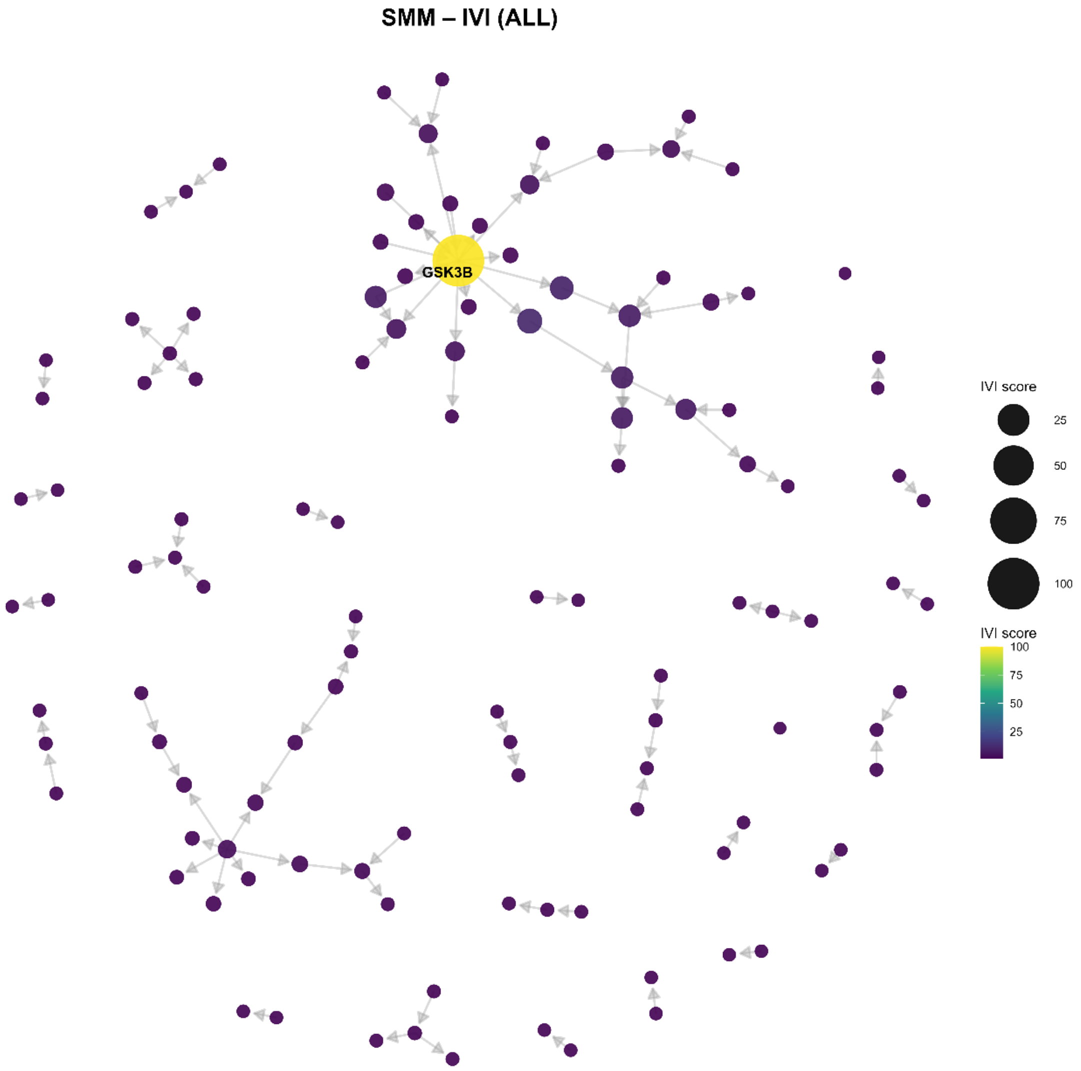

Figure S9: SMM-Specific Network Comparison Based on IVI (ALL). This figure presents the SMM-stage network with nodes sized and coloured according to their outgoing Influence Value Index (all_IVI), calculated using the Influential R package. Larger node sizes and more intense colours indicate stronger outgoing influence within the network. For clarity, only genes with all_IVI > 25 are labelled.

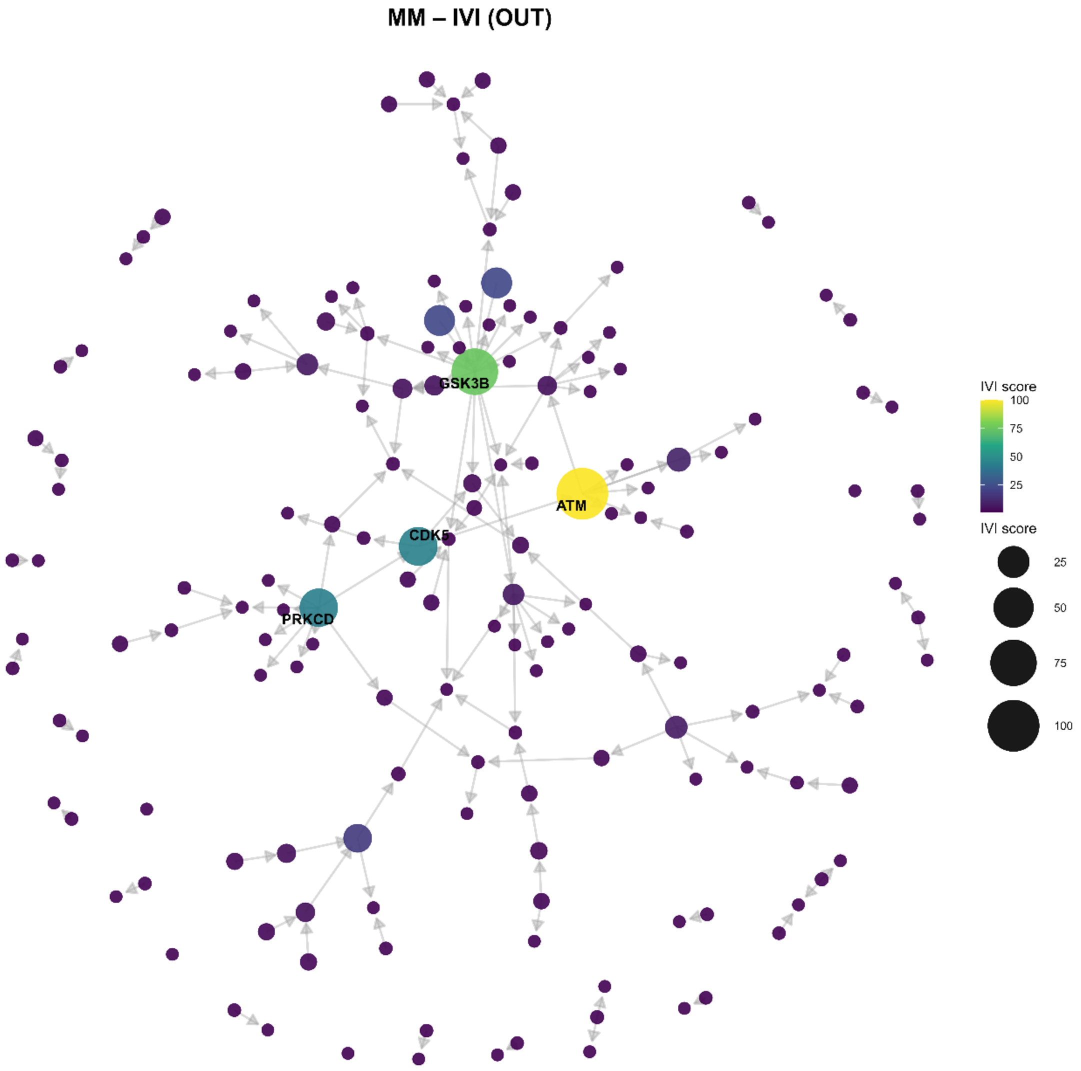

Figure S10: MM-Specific Network Comparison Based on IVI (OUT). This figure presents the MM-stage network with nodes sized and coloured according to their outgoing Influence Value Index (out_IVI), calculated using the Influential R package. Larger node sizes and more intense colours indicate stronger outgoing influence within the network. For clarity, only genes with out_IVI > 25 are labelled.

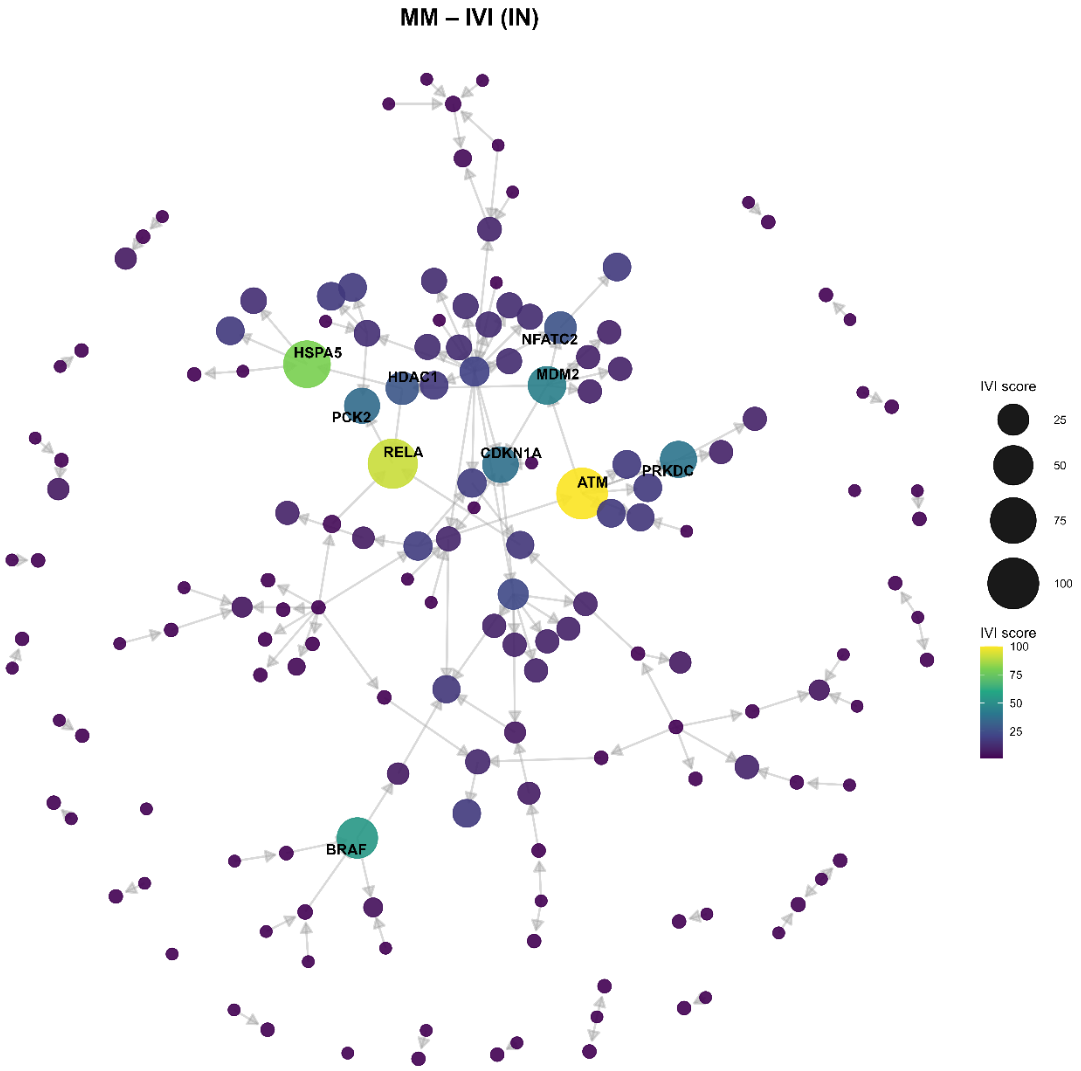

Figure S11: MM-Specific Network Comparison Based on IVI (IN). This figure presents the MM-stage network with nodes sized and coloured according to their outgoing Influence Value Index (in_IVI), calculated using the Influential R package. Larger node sizes and more intense colours indicate stronger outgoing influence within the network. For clarity, only genes with in_IVI > 25 are labelled.

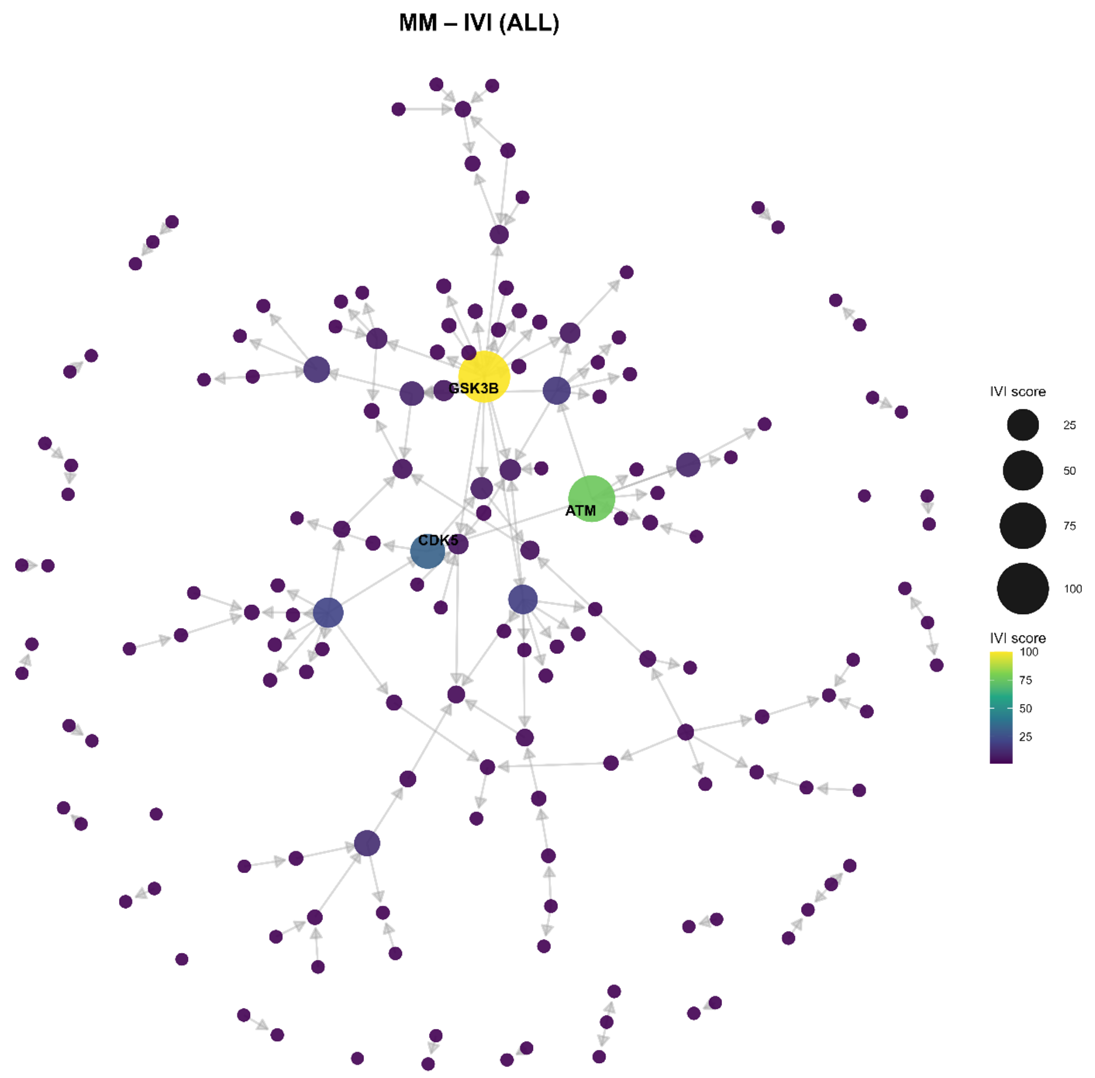

Figure S12: MM-Specific Network Comparison Based on IVI (ALL). This figure presents the MM-stage network with nodes sized and coloured according to their outgoing Influence Value Index (all_IVI), calculated using the Influential R package. Larger node sizes and more intense colours indicate stronger outgoing influence within the network. For clarity, only genes with all_IVI > 25 are labelled.
